# The impact of inflammation on the incidence of different pathological types of lung cancer: the Kailuan study

**DOI:** 10.3389/fonc.2026.1778163

**Published:** 2026-04-27

**Authors:** Songlin Li, Shuohua Chen, Qian Zhang, Xinhong Zhang, Jie Liu, Hui Zhao, Yingzhe Dai, Gang Chen

**Affiliations:** 1 Department of Respiratory and Critical Care Medicine, The Third Hospital of Hebei Medical University, Shijiazhuang, Hebei, China; 2 Department of Respiratory and Critical Care Medicine, Tangshan Gongren Hospital, Tangshan, Hebei, China; 3 Department of Cardiology, Kailuan General Hospital, Tangshan, Hebei, China; 4 Computed Tomography (CT) Room, Tangshan Gongren Hospital, Tangshan, Hebei, China; 5 Department of Critical Care Medicine, Tangshan Gongren Hospital, Tangshan, Hebei, China

**Keywords:** epidemiology, inflammation, lung cancer, pathological types, prospective study

## Abstract

**Background:**

Chronic inflammation drives lung cancer pathogenesis; however, evidence on its association with incident risk across pathological subtypes remains limited.

**Objective:**

To evaluate associations of 15 inflammatory indicators with long-term and 10-year risk of overall and subtype specific lung cancer.

**Methods and results:**

This study included 99, 925 participants from the Kailuan cohort (mean follow-up, 14.85 years). During follow-up, 1, 804 incident lung cancer cases were identified. After multivariable adjustment and false discovery rate correction, several inflammatory markers, particularly the novel composite indices SIRI and AISI, were significantly associated with increased overall lung cancer risk. Per 1 Z-score increase, white blood cell count (HR = 1.112, 95% CI: 1.064–1.163), monocytes (HR = 1.060, 95% CI: 1.033–1.087), lymphocytes (HR = 1.050, 95% CI: 1.014–1.087), neutrophils (HR = 1.083, 95% CI: 1.037–1.130), CLR (HR = 1.031, 95% CI: 1.006–1.058), SIRI (HR = 1.536, 95% CI: 1.183–1.995), and AISI (HR = 1.070, 95% CI: 1.023–1.119) showed significant associations. For 10-year risk, similar associations were observed, with SIRI and AISI increasing risk by 75.7% (HR = 1.757, 95% CI: 1.350–2.278) and 9.0% (HR = 1.090, 95% CI: 1.029–1.154), respectively. Subtype analyses revealed significant positive associations for squamous cell carcinoma (e.g., SIRI: HR = 1.782, 95% CI: 1.352–2.350) and other pathological types (e.g., SIRI: HR = 1.638, 95% CI: 1.263–2.125) but not for adenocarcinoma or small cell lung cancer. Stratified analyses showed effect modification by age for SIRI in overall lung cancer (stronger in participants aged ≥60 years), and by sex, age, and smoking status for multiple markers in subtype-specific analyses. SIRI showed the strongest effects across all analyses.

**Conclusion:**

Chronic inflammation is differentially associated with lung cancer risk by subtype; SIRI and AISI are independent risk factors, with SIRI showing the strongest effects, particularly for squamous cell carcinoma, in individuals aged ≥ 60 years, and these associations are modified by sex and smoking status.

## Introduction

Lung cancer remains the leading cause of cancer-related mortality globally. GLOBOCAN 2022 estimates indicate 2.48 million new cases (12.4% of total cancer incidence) and 1.81 million deaths (18.7% of cancer deaths) annually (1). Despite advances with targeted therapies and immune checkpoint inhibitors in selected subgroups, the 5-year survival rate at the population level remains below 20% (2), underscoring the critical need for effective prevention and early detection strategies. Therefore, developing effective prevention strategies is critical for improving outcomes.

Chronic systemic inflammation is a well-established driver of lung cancer pathogenesis and progression, highlighting its translational significance (3). Routine blood test-derived inflammatory indicators, including high-sensitivity C-reactive protein (hs-CRP) (4, 5), white blood cell count (WBC) (6, 7), the neutrophil-to-lymphocyte ratio (NLR) (8), and the platelet-to-lymphocyte ratio (PLR) (9), are associated with lung cancer risk and prognosis. Novel composite indices integrating multi-dimensional pathophysiology, such as the lymphocyte-to-high-density lipoprotein cholesterol ratio (LHR) (10) and C-reactive protein-to-lymphocyte ratio (CLR) (11, 12), show potentially stronger predictive value for incidence and prognosis.

However, significant knowledge gaps persist. Existing studies typically focus on single pathological subtypes or aggregate all lung cancers, lacking systematic comparisons of differential inflammatory effects across major subtypes [adenocarcinoma (LUAD), squamous cell carcinoma (LUSC), and small cell lung cancer (SCLC)] within a unified cohort. Studies have shown that an elevated systemic inflammation response index (SIRI) correlates with increased mortality in non-small cell lung cancer (NSCLC) (13) and an elevated aggregate index of systemic inflammation (AISI) associates with mortality across cancers broadly (14). However, the associations of these indices with incident lung cancer risk, particularly when stratified by subtype, remain unexplored.

To address these gaps, we leveraged the large-scale prospective Kailuan cohort to comprehensively assess associations of 15 inflammatory indicators, including SIRI and AISI, with incident lung cancer risk. This study specifically aims to elucidate differential associations across major pathological subtypes (LUAD, LUSC, and SCLC), providing novel evidence for subtype specific prevention strategies.

## Materials and methods

### Study subjects

The Kailuan Study (ChiCTR2000029767) is a prospective cohort established within the company-based community of the Kailuan Group, a large state-owned coal mining and allied industries enterprise in Tangshan, China. The study rationale, design, and baseline examinations have been described in detail previously (15–17). Briefly, the initial health examinations were conducted at Kailuan General Hospital and its affiliated medical institutions during 2006–2007, targeting both active and retired employees of the group. Systematic health examinations have since been conducted biennially, with the eighth follow-up round completed by December 31, 2022, resulting in a total of 188, 573 participants in the study cohort. The study protocol was approved by the Ethics Committee of Kailuan General Hospital, and written informed consent was obtained from all participants in accordance with the Declaration of Helsinki.

The Kailuan cohort predominantly comprises industrial workers, reflecting the male-dominated workforce of the parent company. This explains the observed sex imbalance (about 80% male) in our study sample, which represents the underlying occupational population. Tangshan is a major industrial center; therefore, participants may have a history of exposure to urban industrial environments. All participants were enrolled from the same company-based community, sharing similar socioeconomic status and healthcare access.

**Figure 1:**
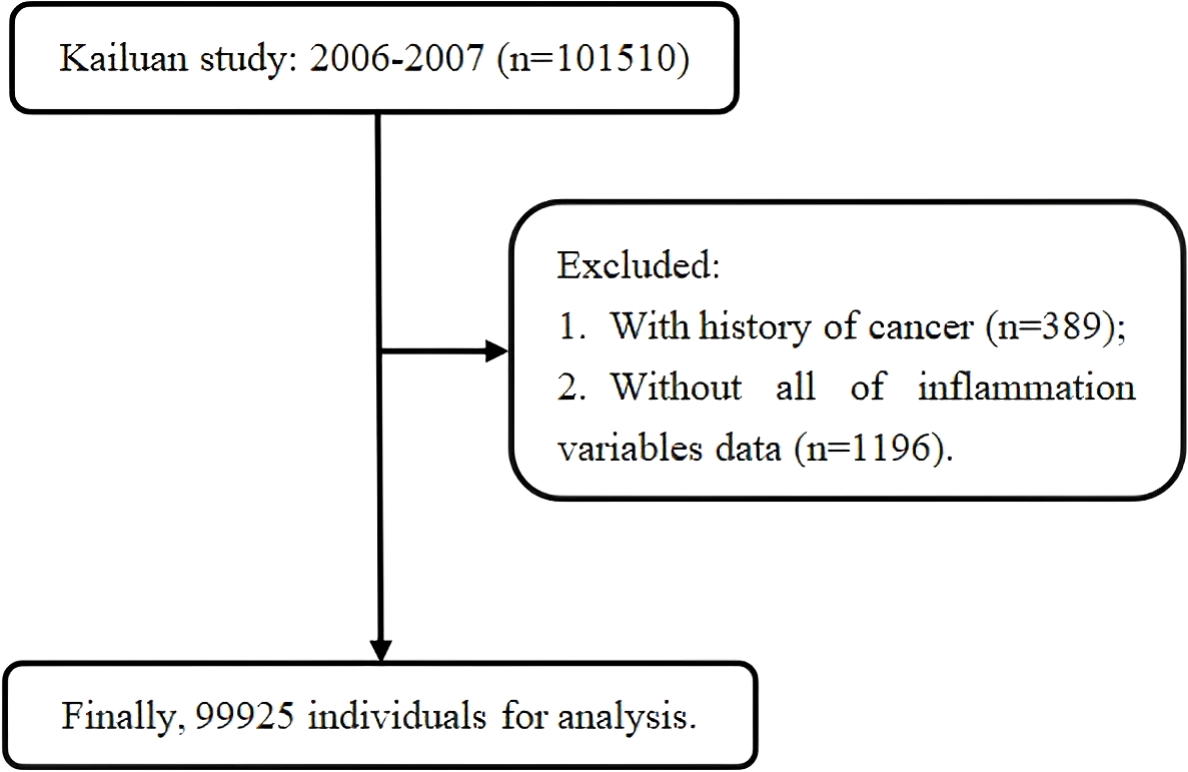
Inclusion and exclusion flowchart.

The participant selection process for this analysis is shown in **Figure 1**. From among the 101, 510 participants who completed the 2006–2007 baseline examination, 389 individuals with a pre-existing cancer diagnosis were excluded. For the primary exposure analysis, an additional 1, 196 individuals with missing data on any of the 15 inflammatory biomarkers were excluded, resulting in a final analytic cohort of 99, 925 individuals.

### Data collection

#### Inflammatory indicators

Fifteen inflammatory indicators were analyzed: 1) Hematological parameters: hs-CRP, WBC, monocytes, lymphocytes, and neutrophils; 2) Cellular ratios: NLR, monocyte-to-lymphocyte ratio (MLR), PLR, and CLR; 3) Systemic inflammation indices: systemic immune-inflammation index (SII = [neutrophils × platelets]/lymphocytes), SIRI [(neutrophils × monocytes)/lymphocytes], and AISI [(neutrophils × monocytes × platelets)/lymphocytes]; 4) Lipid-related ratios: monocyte-to-HDL-C ratio (MHR), neutrophil-to-HDL-C ratio (NHR), and LHR. All indicators were standardized as Z-scores using the formula Z = (X - μ)/σ, where X represents the original value, μ the sample mean, and σ the standard deviation.

#### Specimen collection and laboratory testing

Fasting venous blood samples collected after overnight fasting for 8–12 h were processed within 2 h. Complete blood count analysis using EDTA-anticoagulated samples was performed on Sysmex XN-9000 analyzers (Sysmex Corporation, Japan). Serum hs-CRP was measured using particle-enhanced immunonephelometry (BN ProSpec system, Siemens Healthineers, Germany). High-density lipoprotein cholesterol (HDL-C) was quantified by direct method on Hitachi 7600–020 analyzers (Hitachi High-Technologies, Japan). The central laboratory implemented blinded testing protocols with routine internal quality control and external quality assessment after established procedures (15–17).

#### Covariates

Using structured questionnaires, trained personnel collected data on demographic characteristics (age and sex), socioeconomic factors (education level categorized as high school or above; income level dichotomized at ≥ 1000 CNY/month), and behavioral factors. Under behavioral factors, smoking status was categorized as never smoked, former smoker, occasional smoker, and daily smoker. A former smoker was defined as an individual who had completely ceased smoking for at least 1 year before assessment. Physician-measured blood pressure followed standardized protocols using mercury sphygmomanometers (Omron HBP-1300 devices after 2014). Hypertension was defined as a systolic blood pressure of ≥ 140 mmHg, a diastolic blood pressure of ≥ 90 mmHg, physician-diagnosed hypertension, or antihypertensive medication use (18). Diabetes mellitus was defined as fasting blood glucose ≥ 7.0 mmol/L, physician-diagnosed diabetes, or glucose-lowering medication use (19). Family history of cancer was defined as any cancer diagnosis in either parent. For covariates with a low missing rate (<5%), missing values were handled using multiple imputation. Body mass index (BMI): Calculated by dividing body weight in kilograms at the time of hospital admission by the square of height in meters at the time of admission.

#### Outcomes

The primary outcome was incident lung cancer (ICD-10: C34) identified through the Kailuan Medical Insurance Database with verification via hospital medical records. Cases were classified into pathological subtypes: adenocarcinoma, squamous cell carcinoma, small cell carcinoma, and other/unspecified. Follow-up continued until lung cancer diagnosis, death from non-lung cancer causes, loss to follow-up, or study termination (December 31, 2023).

### Statistical analysis

Continuous variables are presented as mean ± standard deviation for normally distributed data or median (interquartile range) for non-normally distributed data, with between-group comparisons performed using ANOVA or Mann-Whitney U tests as appropriate. Categorical variables are expressed as frequencies (percentages) and compared using χ² or Fisher’s exact tests.

Multivariable Cox proportional hazards regression models were used to evaluate associations between inflammatory indicators and long-term/10-year incident lung cancer risk (overall and by pathological subtype); subdistribution hazard ratios (HRs) and 95% confidence intervals (CIs) were calculated using Fine-Gray competing risk models for subtypes subject to competing risks. All models were adjusted for sex, age, smoking status, education, income, hypertension, diabetes, BMI, and family history of cancer. Based on primary findings, we conducted analyses stratified by sex, age (<60 vs. ≥ 60 years), and smoking status to test subgroup effects. An interaction *P* value of <0.1 was considered indicative of interaction significance.

We further performed a series of sensitivity analyses to assess the robustness of our results: (a) excluding participants diagnosed with lung cancer within 12 months of enrollment to mitigate potential lead-time bias; (b) calculating the cumulative value of each inflammatory marker using three repeated measurements (collected in 2006, 2008, and 2010) based on the trapezoidal rule: cumulative inflammation = (I_2006_ + I_2008_)/2 × (t_2008_ − t_2006_) + (I_2008_ + I_2010_)/2 × (t_2010_ − t_2008_), with these cumulative values transformed into Z-scores and examined for their associations with the outcome; and (c) categorizing all inflammatory markers according to clinically meaningful cutoffs—WBC (<4× 10^9^/L, 4–10 × 10^9^/L, and ≥10 × 10^9^/L), monocytes (<0.1 × 10^9^/L, 0.1–0.6 × 10^9^/L, and ≥0.6 × 10^9^/L), lymphocytes (<1.1 × 10^9^/L, 1.1–3.2 × 10^9^/L, and ≥3.2 × 10^9^/L), and neutrophils (<1.8 × 10^9^/L, 1.8–6.3 × 10^9^/L, and ≥6.3× 10^9^/L)—and tertiles for MLR, CLR, SIRI, AISI, and MHR, then re-evaluating their associations with the outcome; and (d) additionally adjusting for baseline hs-CRP and WBC levels when they were not the primary exposure of interest, to evaluate whether the associations of other inflammatory markers were independent of these two core inflammatory biomarkers.

All analyses were performed using SAS 9.4 (SAS Institute Inc.) with statistical significance defined as a two-tailed *P* value of <0.05. To control the false-positive rate potentially induced by multiple comparisons, we applied the Benjamini–Hochberg false discovery rate correction to the hypothesis tests related to the associations between biomarkers and outcomes (overall and each subtype of lung cancer) in the main analysis. The corrected *P* values (FDR P) were used to determine statistical significance, with the significance level set at <0.05.

### Assessment of multicollinearity

Given the mathematical interdependence among some inflammatory indicators, multicollinearity was assessed in two scenarios relevant to our analysis. First, variance inflation factors (VIFs) were calculated for a model including all 15 inflammatory indicators simultaneously. As expected, this revealed high multicollinearity (VIFs > 5 for most indices), confirming their non-independence as concurrent exposures. However, in our primary Cox regression models, each inflammatory indicator was analyzed separately as the primary exposure variable with the predefined covariates (e.g., age, sex, and smoking status). VIFs calculated for each of these final individual models were all below the conventional threshold of 5, indicating no substantial multicollinearity between the single inflammatory variable of interest and the adjustment covariates. Therefore, the hazard ratios from our main analyses are not biased by multicollinearity.

## Results

### Baseline characteristics of study participants

A cohort of 99, 925 participants was analyzed to investigate the association between inflammatory indicators and incident lung cancer. After a mean follow-up period of 14.85 ± 3.07 years, 1, 804 individuals developed lung cancer (cumulative incidence: 1.33%), including 317 LUADs, 215 LUSCs, 138 SCLCs, and 1, 134 cases of other pathological subtype. This latter category is heterogeneous, comprising both rare subtype and many cases historically classified as non-specific non-small cell lung cancer. The cohort had a mean age of 51.85 ± 12.67 years and comprised 79, 824 men (79.8%). Compared to the non-cancer group, cancer group participants were significantly older and showed significantly elevated levels of BMI, hs-CRP, WBC, monocytes, neutrophils, CLR, SIRI, and AISI. This group also had a significantly higher proportion of males, individuals with hypertension, those reporting a family history of cancer, daily smoking, and individuals with higher education level (all, *P* < 0.05; **Table 1**).

**Table 1:** Baseline characteristics of patients with and without lung cancer.

Variables	Total (N = 999, 25)	Non-lung cancer (N = 98, 121)	Lung cancer (N = 1, 804)	P
Age, years	51.85 ± 12.67	51.74 ± 12.69	57.55 ± 10.31	<0.001
Women	20101(20.12)	19900(20.28)	201(11.14)	<0.001
Men	79824(79.88)	78221(79.72)	1603(88.86)	
High school or above, n(%)	19877(19.89)	19654(20.03)	223(12.36)	<0.001
Income>1000 yuan/moth, n(%)	93455(93.53)	91749(93.51)	1706(94.57)	0.069
Smoking status, n(%)				<0.001
Never smoking	60697(60.74)	59771(60.92)	926(51.33)	
Former smoking	5741(5.75)	5624(5.73)	117(6.49)	
Occasional smoking	3476(3.48)	3446(3.51)	30(1.66)	
Daily smoking	30011(30.03)	29280(29.84)	731(40.52)	
Family history of cancer, n(%)	4616(4.62)	4513(4.60)	103(5.71)	0.026
Hypertension, n(%)	43842(43.87)	42985(43.81)	857(47.51)	0.002
Diabetes, n(%)	9114(9.12)	8946(9.12)	168(9.31)	0.775
Body mass index, kg/m^2^	25.05 ± 3.48	25.05 ± 3.48	24.71 ± 3.40	<0.001
Hs-CRP, mg/L	0.80(0.30-2.10)	0.80(0.30-2.10)	0.95(0.35-2.41)	0.006
White blood cell, 10^3^/μL	6.62 ± 1.74	6.62 ± 1.74	6.80 ± 1.81	<0.001
Monocytes, 10^3^/μL	0.42 ± 0.35	0.42 ± 0.35	0.46 ± 0.61	0.001
Lymphocytes, 10^3^/μL	2.32 ± 0.95	2.32 ± 0.95	2.37 ± 0.95	0.063
Neutrophils, 10^3^/μL	3.92 ± 1.37	3.92 ± 1.37	4.05 ± 1.41	<0.001
Platelet, 10^9^/L	200.00(168.00-237.00)	200.00(168.00-237.00)	198.00(163.00-235.00)	0.016
HDL-C, mmol/L	1.55 ± 0.40	1.55 ± 0.40	1.55 ± 0.41	0.772
NLR	1.86 ± 1.48	1.86 ± 1.49	1.87 ± 0.86	0.626
MLR	0.17(0.13-0.22)	0.17(0.13-0.22)	0.17(0.13-0.23)	0.471
PLR	90.42(72.07-113.16)	90.43(72.11-113.18)	87.66(68.57-111.18)	0.507
CLR	0.36(0.14-1.01)	0.36(0.13-1.01)	0.42(0.16-1.23)	<0.001
SII	335.19(247.68-451.86)	335.16(247.78-451.56)	336.67(242.53-464.88)	0.999
SIRI	0.62(0.41-0.92)	0.62(0.41-0.92)	0.65(0.43-0.97)	0.007
AISI	152.37 ± 117.68	152.24 ± 117.53	158.91 ± 125.13	0.021
MHR	0.24(0.17-0.35)	0.24(0.17-0.35)	0.25(0.17-0.36)	0.367
LHR	1.66 ± 2.76	1.66 ± 2.78	1.65 ± 1.08	0.857
NHR	2.79 ± 2.98	2.79 ± 3.00	2.81 ± 1.39	0.754

NLR, neutrophil to lymphocyte ratio; MLR, monocyte to lymphocyte ratio; PLR, platelet to lymphocyte ratio; CLR, hs-CRP to lymphocyte ratio; SII, neutrophil*platelet/lymphocyte; SIRI, neutrophil*monocyte/lymphocyte; AISI, neutrophil*monocyte*platelet/lymphocyte; MHR, monocyte/high-density lipoprotein cholesterol; LHR, lymphocyte/high-density lipoprotein cholesterol; NHR, neutrophil/high-density lipoprotein cholesterol.

The participants’ baseline characteristics in total and by the incident of lung cancer were presented as mean ± standard deviation (SD) and median with interquartile range (IQR) for normally and non-normally distributed continuous variables, respectively, and as numbers with percentages for categorical variables. The distinctions in attributes among groups were scrutinized using the Chi-squared (χ2) test or Fisher’s Exact Test for categorical variables, and the Kruskal-Wallis test for continuous variables, respectively.

Significant differences in baseline characteristics across pathological lung cancer subtypes are detailed in **Supplementary Table 1.1** (all, *P* < 0.05). These differences encompassed age, sex, education level, smoking status, family history of cancer, hypertension, and levels of BMI, hs-CRP, WBC, monocytes, neutrophils, platelets, CLR, SIRI, and AISI. Specifically, patients with LUAD presented with the youngest age, the highest proportion of females, the highest incidence of a family history of cancer, the lowest neutrophil levels, and the highest platelet levels. Patients with LUSC had the highest proportion of men and daily smoking, along with the highest levels of BMI, WBC and monocytes and the highest AISI. Patients with SCLC had the lowest proportion of individuals with high school or higher education, the highest hypertension, and the highest SIRI. Finally, patients with other pathological subtype had the highest hs-CRP and CLR levels.

### Inflammation and the long-term risk of overall lung cancer

Multivariable-adjusted Cox regression analysis showed significant positive associations of WBC, monocytes, lymphocytes, neutrophils, CLR, SIRI, and AISI and the overall long-term lung cancer risk after Benjamini–Hochberg FDR correction (all, FDR *P* < 0.05). For each 1 Z-score increment, the hazard increases were 11.2% (HR: 1.112, 95% CI: 1.064–1.163), 6.0% (HR: 1.060, 95% CI: 1.033–1.087), 5.0% (HR: 1.050, 95% CI: 1.014–1.087), 8.3% (HR: 1.083, 95% CI: 1.037–1.130), 3.1% (HR: 1.031, 95% CI: 1.006–1.058), 53.6% (HR: 1.536, 95% CI: 1.183–1.995), and 7.0% (HR: 1.070, 95% CI: 1.023–1.119), respectively (**Table 2**, **Supplementary Table 2.1**). Among the markers identified to be significantly associated with the overall lung cancer risk in the main analysis, the WBC remained statistically significant in all four sensitivity analyses. These analyses included the exclusion of early diagnoses (**Supplementary Table 3.1**), cumulative exposure modeling (**Supplementary Table 3.2**), categorical cutoffs (**Supplementary Table 3.3**), and additional adjustment for hs-CRP and WBC (**Supplementary Table 3.4**).

**Table 2:** The HR(95% CI) between inflammation and the incidence risk of lung cancer.

Variables	Lung cancer	Lung squamous cell carcinomas	Lung adenocarcinoma	Small cell lung cancer	Other pathological subtype of lung cancer
Hs-CRP	1.027(0.982-1.074)	1.124(0.990-1.277)	0.910(0.806-1.029)	0.925(0.768-1.113)	1.044(0.991-1.100)
WBC	1.112(1.064-1.163)	1.092(0.948-1.257)	1.086(0.975-1.209)	1.075(0.915-1.264)	1.123(1.065-1.185)
Monocytes	1.060(1.033-1.087)	1.101(1.061-1.143)	1.017(0.929-1.114)	1.045(0.999-1.094)	1.034(1.007-1.062)
Lymphocytes	1.050(1.014-1.087)	1.035(0.956-1.120)	0.997(0.916-1.086)	1.075(0.981-1.178)	1.058(1.027-1.091)
Neutrophils	1.083(1.037-1.130)	1.065(0.939-1.208)	1.078(0.981-1.184)	1.066(0.916-1.240)	1.085(1.031-1.142)
NLR	0.993(0.934-1.055)	1.013(0.960-1.068)	0.992(0.899-1.094)	0.948(0.755-1.190)	0.989(0.928-1.055)
MLR	1.012(0.985-1.039)	1.026(1.014-1.038)	1.002(0.962-1.043)	0.946(0.793-1.130)	1.010(0.995-1.026)
PLR	0.982(0.863-1.118)	0.931(0.592-1.464)	1.006(0.995-1.017)	0.999(0.899-1.111)	0.906(0.709-1.157)
CLR	1.031(1.006-1.058)	1.065(1.037-1.094)	1.028(0.959-1.102)	0.664(0.446-0.990)	1.018(0.992-1.044)
SII	1.004(0.971-1.038)	1.009(0.977-1.042)	1.005(0.989-1.020)	0.993(0.845-1.167)	1.003(0.988-1.019)
SIRI	1.536(1.183-1.995)	1.782(1.352-2.350)	0.530(0.093-3.012)	0.968(0.435-2.154)	1.638(1.263-2.125)
AISI	1.070(1.023-1.119)	1.146(1.012-1.298)	1.062(0.956-1.180)	1.094(0.936-1.278)	1.050(0.993-1.111)
MHR	1.014(0.992-1.035)	1.027(1.015-1.039)	0.904(0.695-1.175)	0.953(0.847-1.072)	1.014(1.001-1.028)
LHR	1.004(0.962-1.048)	0.988(0.905-1.078)	0.888(0.694-1.135)	1.012(0.968-1.057)	1.011(0.996-1.027)
NHR	1.008(0.974-1.043)	1.003(0.945-1.065)	0.987(0.916-1.065)	1.009(0.966-1.054)	1.010(0.996-1.024)

NLR, neutrophil to lymphocyte ratio; MLR, monocyte to lymphocyte ratio; PLR, platelet to lymphocyte ratio; CLR, hs-CRP to lymphocyte ratio; SII, neutrophil*platelet/lymphocyte; SIRI, neutrophil*monocyte/lymphocyte; AISI, neutrophil*monocyte*platelet/lymphocyte; MHR, monocyte/high-density lipoprotein cholesterol; LHR, lymphocyte/high-density lipoprotein cholesterol; NHR, neutrophil/high-density lipoprotein cholesterol.

Model adjusted for age, sex, current smoking, education level, income level, hypertension, diabetes, body mass index and family history of cancer.

Stratified analysis indicated that the associations of hs-CRP, NLR, and SIRI with overall lung cancer risk varied by age (*P*_interaction_ *<*0.1). Further analysis showed that only the risk difference for SIRI in the age subgroup was statistically significant. Specifically, among individuals aged ≥ 60 years, each 1 Z-score increase in SIRI was significantly associated with a higher risk of overall lung cancer (HR = 2.664, 95% CI: 1.858–3.822), whereas there was no significant increase in risk among those aged <60 years (HR = 1.220, 95% CI: 0.760–1.959). This finding suggests that SIRI is associated with a higher risk of overall lung cancer in older adults. No differences were noted based on sex or smoking status (**Table 3**).

**Table 3:** Stratification analysis: the HR(95% CI) between inflammation and the incidence risk of lung cancer.

Variables	Sex		Age		Smoking status			
	Men	Women	≥60 years old	<60 years old	Never smoking	Former smoking	Occasional smoking	Daily smoking
Hs-CRP	1.047(1.000-1.097)	0.823(0.693-0.977)	1.031(0.963-1.104)	1.032(0.972-1.096)	1.016(0.958-1.078)	1.016(0.839-1.229)	1.165(0.835-1.625)	1.042(0.965-1.125)
WBC	1.111(1.059-1.164)	1.124(0.972-1.300)	1.084(1.005-1.170)	1.133(1.072-1.199)	1.089(1.021-1.162)	1.078(0.894-1.299)	1.245(0.898-1.726)	1.136(1.061-1.217)
Monocytes	1.063(1.037-1.089)	0.979(0.831-1.154)	1.074(1.035-1.115)	1.049(1.013-1.086)	1.078(1.040-1.118)	0.942(0.703-1.261)	1.199(1.002-1.436)	1.045(1.004-1.088)
Lymphocytes	1.053(1.016-1.091)	1.016(0.890-1.160)	1.060(1.001-1.123)	1.041(0.995-1.089)	1.034(0.984-1.087)	1.040(0.899-1.204)	1.206(1.030-1.412)	1.061(1.002-1.123)
Neutrophils	1.079(1.030-1.130)	1.105(0.980-1.246)	1.050(0.973-1.134)	1.105(1.050-1.162)	1.080(1.016-1.148)	1.074(0.888-1.300)	1.098(0.755-1.597)	1.085(1.016-1.158)
NLR	0.971(0.897-1.050)	1.022(0.972-1.075)	0.920(0.811-1.044)	1.022(0.980-1.067)	1.001(0.948-1.058)	1.057(0.798-1.399)	0.565(0.228-1.398)	0.975(0.861-1.103)
MLR	1.013(0.979-1.048)	1.009(0.966-1.055)	1.021(0.985-1.058)	1.004(0.955-1.055)	1.013(0.986-1.040)	0.358(0.018-7.111)	1.024(0.404-2.595)	1.030(0.891-1.190)
PLR	0.913(0.767-1.088)	1.002(0.963-1.042)	0.955(0.737-1.236)	0.997(0.899-1.105)	1.002(0.963-1.043)	0.945(0.537-1.661)	0.248(0.042-1.459)	0.849(0.636-1.134)
CLR	1.032(1.006-1.058)	0.972(0.788-1.199)	1.021(0.978-1.066)	1.044(1.016-1.073)	1.011(0.956-1.068)	1.130(1.062-1.203)	0.792(0.372-1.687)	1.040(1.005-1.077)
SII	1.007(0.948-1.069)	1.002(0.958-1.048)	0.992(0.887-1.109)	1.016(0.976-1.058)	1.005(0.973-1.039)	1.106(0.818-1.496)	0.833(0.238-2.917)	0.988(0.825-1.183)
SIRI	1.557(1.137-2.132)	1.496(0.921-2.429)	2.664(1.858-3.822)	1.220(0.760-1.959)	1.517(1.158-1.987)	–	1.206(0.023-64.133)	1.567(0.600-4.090)
AISI	1.071(1.022-1.122)	1.051(0.904-1.221)	1.006(0.929-1.090)	1.113(1.055-1.175)	1.096(1.029-1.167)	0.900(0.720-1.126)	1.236(0.926-1.648)	1.058(0.988-1.132)
MHR	1.022(0.990-1.056)	1.006(0.961-1.054)	1.084(1.018-1.154)	1.004(0.960-1.051)	1.013(0.990-1.035)	0.618(0.067-5.749)	1.074(0.666-1.733)	1.029(0.895-1.184)
LHR	1.006(0.959-1.055)	0.999(0.894-1.116)	1.008(0.952-1.067)	1.001(0.940-1.066)	0.994(0.920-1.073)	0.947(0.590-1.518)	1.033(0.842-1.267)	1.018(0.960-1.078)
NHR	1.005(0.967-1.046)	1.035(0.939-1.140)	1.004(0.951-1.060)	1.016(0.970-1.064)	1.002(0.950-1.057)	0.969(0.727-1.291)	0.990(0.456-2.149)	1.027(0.966-1.092)

NLR, neutrophil to lymphocyte ratio; MLR, monocyte to lymphocyte ratio; PLR, platelet to lymphocyte ratio; CLR, hs-CRP to lymphocyte ratio; SII, neutrophil*platelet/lymphocyte; SIRI, neutrophil*monocyte/lymphocyte; AISI, neutrophil*monocyte*platelet/lymphocyte; MHR, monocyte/high-density lipoprotein cholesterol; LHR, lymphocyte/high-density lipoprotein cholesterol; NHR, neutrophil/high-density lipoprotein cholesterol.

Model adjusted for age (exclude age stratified analysis), sex (exclude sex stratified analysis), smoking status (exclude smoking stratified analysis), education level, income level, hypertension, diabetes, body mass index and family history of cancer.

### Inflammation and the long-term risk of LUSC

In LUSC, after FDR correction, each 1 Z-score increase in monocyte, MLR, CLR, SIRI, and MHR was significantly associated with elevated long-term risk (all, FDR *P* < 0.05). The HR (95% CI) values were 1.101 (1.061–1.143), 1.026 (1.014–1.038), 1.065 (1.037–1.094), 1.782 (1.352–2.350), and 1.027 (1.015–1.039), respectively (**Table 2**). Not all inflammatory markers associated with the risk of lung squamous cell carcinoma maintained consistent results in all sensitivity analyses (**Supplementary Tables 3**-**6**, corresponding to the four sensitivity analyses: exclusion of early diagnoses, cumulative exposure modeling, categorical cutoffs, and additional adjustment for hs-CRP and WBC).

Stratified analyses revealed effect modification by sex, age, and smoking status in the associations between inflammatory markers and the risk of LUSC. For sex differences, significant interactions were observed for WBC, lymphocyte, and MHR with LUSC risk. The positive association of MHR was statistically significant in both sexs but more pronounced in men (HR = 1.038, 95% CI: 1.016–1.061) than in women (HR = 1.012, 95% CI: 1.002–1.021). Regarding age differences, the associations of hs-CRP, monocytes, SIRI, and MHR with LUSC risk varied significantly by age: hs-CRP (HR = 1.183, 95% CI: 1.029–1.360) and monocytes (HR = 1.051, 95% CI: 1.018–1.084) were significantly associated with increased risk only among individuals aged <60 years, with no significant effects in those aged ≥60 years; conversely, SIRI showed a markedly stronger association in older adults (≥60 years: HR = 3.533, 95% CI: 2.019–6.181) compared to younger individuals (<60 years: HR = 1.364, 95% CI: 1.036–1.797), and MHR was positively associated in both age groups but was more pronounced in the elderly (≥60 years: HR = 1.136, 95% CI: 1.049–1.231) than in younger individuals (HR = 1.023, 95% CI: 1.008–1.038). Regarding smoking status, a significant interaction was found for monocyte count, with positive associations observed among never smokers (HR = 1.146, 95% CI: 1.115–1.178), former smokers (HR = 1.137, 95% CI: 1.003–1.289), and daily smokers (HR = 1.080, 95% CI: 1.037–1.125), with the highest risk estimates in never and former smokers; no significant association was detected in occasional smokers (HR = 0.299, 95% CI: 0.050–1.789; **Table 4**).

**Table 4:** Stratification analysis: the HR(95% CI) between inflammation and the incidence risk of lung squamous cell carcinomas.

Variables	Sex		Age		Smoking status			
	Men	Women	≥60 years old	<60 years old	Never smoking	Former smoking	Occasional smoking	Daily smoking
Hs-CRP	1.133(0.998-1.287)	0.613(0.307-1.223)	0.977(0.751-1.272)	1.183(1.029-1.360)	1.213(1.029-1.431)	0.496(0.208-1.180)	0.821(0.361-1.866)	1.047(0.852-1.287)
WBC	1.075(0.933-1.239)	1.794(0.926-3.475)	1.063(0.763-1.479)	1.100(0.939-1.289)	1.056(0.838-1.329)	0.688(0.340-1.394)	1.745(1.394-2.184)	1.113(0.921-1.346)
Monocytes	1.100(1.059-1.143)	1.205(1.109-1.308)	1.148(1.125-1.171)	1.064(1.026-1.103)	1.146(1.115-1.178)	1.137(1.003-1.289)	0.299(0.050-1.789)	1.080(1.037-1.125)
Lymphocytes	1.020(0.930-1.119)	1.198(1.137-1.262)	1.011(0.792-1.292)	1.033(0.946-1.128)	0.982(0.830-1.163)	0.691(0.176-2.717)	1.278(1.103-1.481)	1.065(0.974-1.164)
Neutrophils	1.063(0.935-1.208)	1.155(0.720-1.853)	1.019(0.750-1.386)	1.080(0.947-1.233)	1.070(0.863-1.328)	0.650(0.187-2.252)	1.390(1.005-1.923)	1.072(0.927-1.240)
NLR	1.016(0.980-1.054)	0.304(0.016-5.786)	0.960(0.667-1.382)	1.033(0.980-1.089)	1.013(0.969-1.058)	1.634(0.711-3.756)	0.912(0.157-5.301)	0.947(0.702-1.280)
MLR	1.029(1.014-1.045)	0.989(0.819-1.195)	1.037(1.012-1.064)	1.020(1.004-1.037)	1.026(1.012-1.040)	1.016(0.987-1.045)	-	1.081(0.991-1.179)
PLR	0.951(0.620-1.458)	0.393(0.007-20.668)	1.004(0.974-1.034)	0.903(0.503-1.620)	0.919(0.517-1.632)	1.354(1.068-1.717)	0.294(0.009-10.098)	0.775(0.334-1.799)
CLR	1.065(1.037-1.094)	0.150(0.012-1.831)	1.062(0.994-1.134)	1.068(1.036-1.100)	1.030(0.965-1.100)	1.254(1.198-1.314)	0.609(0.102-3.643)	1.078(1.036-1.122)
SII	1.016(0.969-1.066)	0.808(0.033-19.788)	0.963(0.538-1.726)	1.016(0.981-1.054)	1.004(0.958-1.053)	1.511(1.083-2.107)	1.951(0.589-6.458)	0.914(0.584-1.430)
SIRI	1.807(1.356-2.408)	1.288(0.638-2.602)	3.533(2.019-6.181)	1.364(1.036-1.797)	1.624(1.118-2.359)	6.799(1.105-41.847)	-	2.327(1.344-4.028)
AISI	1.138(1.001-1.294)	1.556(1.095-2.211)	1.010(0.803-1.271)	1.183(1.028-1.362)	1.220(0.991-1.501)	0.721(0.370-1.404)	0.958(0.304-3.022)	1.107(0.957-1.281)
MHR	1.038(1.016-1.061)	1.012(1.002-1.021)	1.136(1.049-1.231)	1.023(1.008-1.038)	1.026(1.013-1.040)	1.097(0.942-1.278)	-	1.078(0.998-1.164)
LHR	0.965(0.813-1.144)	1.034(1.018-1.050)	1.015(0.948-1.087)	0.977(0.854-1.118)	0.940(0.701-1.261)	0.715(0.044-11.537)	1.021(0.975-1.070)	1.006(0.927-1.092)
NHR	0.996(0.914-1.085)	1.076(1.018-1.137)	0.998(0.851-1.171)	1.011(0.951-1.075)	0.998(0.917-1.085)	1.009(0.833-1.221)	1.505(1.187-1.907)	1.004(0.871-1.158)

NLR, neutrophil to lymphocyte ratio; MLR, monocyte to lymphocyte ratio; PLR, platelet to lymphocyte ratio; CLR, hs-CRP to lymphocyte ratio; SII, neutrophil*platelet/lymphocyte; SIRI, neutrophil*monocyte/lymphocyte; AISI, neutrophil*monocyte*platelet/lymphocyte; MHR, monocyte/high-density lipoprotein cholesterol; LHR, lymphocyte/high-density lipoprotein cholesterol; NHR, neutrophil/high-density lipoprotein cholesterol.

Model adjusted for age (exclude age stratified analysis), sex (exclude sex stratified analysis), current smoking (exclude smoking stratified analysis), education level, income level, hypertension, diabetes, body mass index and family history of cancer.

### Inflammation and the long-term risk of LUAD

Multivariable Cox regression showed no significant association between any of the studied inflammatory markers and overall LUAD risk after FDR correction (all, FDR *P* > 0.05) (**Table 2**, **Supplementary Table 2**). In sensitivity analyses, after excluding specific populations or applying different statistical approaches, the associations between certain inflammatory markers and lung adenocarcinoma risk changed, with some markers showing significantly increased risks. After excluding participants diagnosed within 1 year, no significant associations were found (**Supplementary Table 3.1**, exclusion of early diagnoses). In sensitivity analysis 2, only CLR was significantly associated with reduced risk (HR = 0.701, 95% CI: 0.543–0.903; **Supplementary Table 3.2**, cumulative exposure modeling). When the inflammatory indicators were grouped, elevated WBC (≥10 × 10^9^/L), monocytes (≥0.6 × 10^9^/L), lymphocytes (≥3.2 × 10^9^/L), and neutrophils (≥6.3 × 10^9^/L) were all associated with increased risk, with neutrophils showing the highest risk (HR = 2.265, 95% CI: 1.099–4.669) (**Supplementary Table 3.3**, categorical cutoffs). With additional adjustment for hs-CRP and WBC, only PLR remained significant (**Supplementary Table 3.4**, additional adjustment for hs-CRP and WBC).

Stratified analyses indicated that the association between monocytes and LUAD risk varied by sex (*P* for interaction < 0.1). However, subsequent subgroup analyses did not show statistically significant associations in either men or women, making meaningful comparisons of risk magnitude between the sexes impossible. Similarly, significant interactions by smoking status were observed for white blood cell and lymphocyte counts in relation to LUAD risk (both, *P* for interaction < 0.1); however, no statistically significant effects were identified in any smoking category during subgroup analysis. No evidence of effect modification by age was detected for any of the inflammatory markers examined (**Supplementary Table 4.1**, ).

### Inflammation and the long-term risk of SCLC

For SCLC, none of the inflammatory markers showed a statistically significant association with long-term risk after FDR correction (all, FDR *P* > 0.05; **Table 2**, **Supplementary Table 2**). In sensitivity analyses, the associations between inflammatory markers and SCLC risk varied across different models. In sensitivity analysis 1 (**Supplementary Table 3**, exclusion of early diagnoses), only CLR was significantly associated with reduced risk. In sensitivity analysis 2 (**Supplementary Table 4**, cumulative exposure modeling), both CLR and NHR showed significant associations, with CLR indicating reduced risk and NHR indicating increased risk. In sensitivity analysis 3 (**Supplementary Table 5**, categorical cutoffs), elevated monocytes (≥0.6 × 10^9^/L) and neutrophils (≥6.3 × 10^9^/L) were associated with increased risk, with neutrophils showing the highest risk. In sensitivity analysis 4 (**Supplementary Table 6**, additional adjustment for hs-CRP and WBC), no significant associations were observed.

Stratified analyses showed that the associations of NLR and SII with the risk of SCLC varied according to sex (both, *P* for interaction < 0.1). However, subgroup analyses did not yield statistically significant results in either sex, despite the significant interaction. Similarly, a significant interaction by age was observed for AISI in relation to SCLC risk (*P* for interaction < 0.1); however, no statistically significant associations were found in either age group during subgroup analysis, preventing further interpretation of age-specific effects. No evidence of effect modification by smoking status was found for any of the inflammatory markers examined (**Supplementary Table 4.2**).

### Inflammation and the long-term risk of other pathological lung cancer types

After FDR correction, each Z-score increase in WBC (HR = 1.123, 95% CI: 1.065–1.185), monocytes (HR = 1.034, 95% CI: 1.007–1.062), lymphocytes (HR = 1.058, 95% CI: 1.027–1.091), neutrophils (HR = 1.085, 95% CI: 1.031–1.142), and SIRI (HR = 1.638, 95% CI: 1.263–2.125) was significantly associated with an increased risk of other pathological lung cancer (all, FDR *P* < 0.05; **Table 2**, **Supplementary Table 2**). In sensitivity analyses, the associations of different inflammatory markers with the risk of other types of lung cancer showed varying degrees of robustness. Among these, SIRI was the most robust and remained significant in all sensitivity analyses. However, other inflammatory markers, including WBC, monocytes, lymphocytes, neutrophils, AISI, LHR, and NHR were significant only in some sensitivity analyses (**Supplementary Tables 3**-**6**, exclusion of early diagnoses, cumulative exposure modeling, categorical cutoffs, and additional adjustment for hs-CRP and WBC). In addition, MHR and CLR may not have been significant in the main analysis but showed significant associations in multiple sensitivity analyses—MHR was significant in the exclusion of diagnoses within 1 year (**Supplementary Table 3**), categorical cutoffs analysis (**Supplementary Table 5**), and additional adjustment for hs-CRP and WBC (**Supplementary Table 6**); CLR was significant in the time-series cumulative value (**Supplementary Table 4**) and category analysis (**Supplementary Table 5**).

Stratified analyses revealed significant effect modification by sex and age in the associations between inflammatory markers and the risk of other lung cancer types. For sex differences, significant interactions were observed for hs-CRP and NHR (both, *P* for interaction < 0.1). In particular, hs-CRP was significantly associated with increased risk in men (HR = 1.063, 95% CI: 1.008–1.122), whereas no such association was noted in women (HR = 0.826, 95% CI: 0.666–1.023), indicating a stronger carcinogenic effect in men. In contrast, NHR showed a significant positive association only in women (HR = 1.049, 95% CI: 1.015–1.084), with no significant effect observed in men (HR = 1.006, 95% CI: 0.989–1.024), suggesting a sex-specific role for NHR in women. Regarding age differences, significant interactions were identified for SIRI and MHR (both, *P* for interaction < 0.1). SIRI was positively associated with risk in both age groups but showed a much stronger effect in participants aged ≥60 years (HR = 2.573, 95% CI: 1.797–3.685) compared to those aged <60 years (HR = 1.317, 95% CI: 1.016–1.708). MHR showed a significant association only among older individuals (≥60 years: HR = 1.082, 95% CI: 1.005–1.166), with no significant effect in the younger group (HR = 0.997, 95% CI: 0.966–1.029), further supporting an age-dependent effect. No evidence of effect modification by smoking status was detected for any of the markers examined in this subtype (**Supplementary Table 4.3**).

### Inflammation and the 10-year risk of lung cancer

In addition, we assessed the 10-year incident risk of lung cancer (**Supplementary Table 5.1**). After applying the Benjamini–Hochberg FDR correction, for overall lung cancer, each 1 Z-score increase in WBC, monocytes, neutrophils, CLR, SIRI, and AISI remained significantly associated with elevated 10-year risk (all, FDR *P* < 0.05). For LUSC, significant positive associations with 10-year risk were observed for monocytes, MLR, CLR, SIRI, and MHR (all, FDR *P* < 0.05). For LUAD and SCLC, no inflammatory markers showed statistically significant associations with 10-year risk after FDR correction (all FDR P > 0.05). For other pathological subtypes of lung cancer, each 1 Z-score increase in WBC, monocytes, lymphocytes, neutrophils, and SIRI was significantly associated with elevated 10-year risk (all, FDR *P* < 0.05).

## Discussion

This study included 99, 925 participants with a mean follow-up period of 14.85 ± 3.07 years. Key findings were as follows: Several inflammatory indicators showed positive associations with lung cancer risk, and the strength of inflammation-lung cancer associations showed heterogeneity across pathological subtypes and sex, age, and smoking status. To our knowledge, this is the first report linking elevated SIRI and AISI with increased incident lung cancer risk.

Our analysis revealed that with each 1 Z-score increase, several inflammatory indicators elevated long-term overall lung cancer risk by 3.1%–53.6% after the Benjamini–Hochberg FDR correction. WBC remained robust in all sensitivity analysis. Previous studies (20–23) have confirmed associations between elevated inflammatory markers (e.g., WBC, CLR, and SII) and increased lung cancer risk. Therese et al. reported that elevated SII and NLR increased lung cancer risk by 69% and 53%, respectively, in the UK Biobank cohort (21). In addition, another UK Biobank study documented a HR of 1.14 (95% CI: 1.08–1.20) for lung cancer per WBC increase (22). Our results are consistent with these findings. Furthermore, we observed that elevated levels of several inflammatory indicators were associated with increased 10-year lung cancer risk across all pathological subtypes. Notably, the magnitude of 10-year risk elevation generally exceeded that of long-term risk. Thus, lung cancer prevention strategies should prioritize assessment and management of both long-term and 10-year risk. In addition to traditional markers (e.g., WBC and monocytes), composite indicators (e.g., SII, NLR, SIRI, AISI) should be incorporated into risk monitoring.

Second, we observed pathological subtype specific associations. Elevated inflammatory indicators (i) significantly increased long-term risk in overall lung cancer, LUSC, and other subtypes, (ii) showed no significant association in LUAD and SCLC. A prior study reported a 55% increased LUSC risk in a group with WBC > 7.86 × 10^9^/L compared with the lowest WBC group (≤ 5.64 × 10^9^/L) (24). However, most inflammation–lung cancer studies focused on patient prognosis or risk stratification (6, 9, 11, 25, 26). To our knowledge, only one study investigated the association between the lymphocyte-to-monocyte ratio (20) and incident lung cancer without pathological subtype analysis. In addition, we identified sex-based heterogeneity; inflammatory elevations increased lung cancer risk by 3.1%-55.6% in males, whereas elevated hs-CRP was associated with a 16.1% risk reduction in females. Although sex interaction was not statistically significant (*P*_interaction_ > 0.05), HR magnitudes suggested stronger associations in males. A previous UK Biobank study also found that WBC > 9.3 × 10^9^/L increased incident lung cancer risk by 195% and 115% in male and female current smokers, respectively (6), which is consistent with our observations. Therefore, precision prevention strategies should account for pathological subtypes and prioritize male populations.

Our study also observed a trend suggesting reduced SCLC risk with higher CLR. These findings appear to contrast with large prospective studies that generally report positive associations between systemic inflammation (including CLR) and lung cancer risk (11, 12, 27, 28). However, this relationship did not persist after FDR correction (FDR P > 0.05), strongly suggesting that it may be due to chance. Therefore, these results should be considered exploratory and require independent validation. Potential explanations for these observations include the following: (1) limited statistical power in subgroup analyses (e.g., only 138 SCLC cases); (2) complex tumor biology in SCLC, where inflammatory markers may reflect treatment response or paraneoplastic effects rather than etiology; and (3) unmeasured confounding specific to women in our cohort. We highlight these findings to underscore the context-dependent nature of inflammatory biomarkers in cancer risk, which may vary by subtype.

To our knowledge, previous studies have not investigated the association of SIRI and AISI with incident lung cancer risk, with limited reports on their prognostic value in cancers like pancreatic, colorectal, breast, and esophageal cancer (29–33). This may be the first report to demonstrate that elevated SIRI significantly increases incident risk in overall lung cancer (HR = 1.536, 95% CI: 1.183–1.995), LUSC (HR = 1.782, 95% CI: 1.352–2.350), and other subtypes (HR = 1.638, 95% CI: 1.263–2.125), with SIRI showing the strongest association among all studied markers. Importantly, these associations persisted in the 10-year risk analysis, with SIRI and AISI elevations increasing 10-year overall lung cancer risk by 75.4% and 7.0%, respectively. Consequently, individuals with elevated SIRI and AISI (particularly SIRI) require heightened vigilance for lung cancer risk, particularly during the next decade of their lives.

Our study has several advantages. First, the large sample size ensured the reliability of our results. The prospective design minimized the potential for recall bias and provided comprehensive data for our analysis. Second, our study offers the first comprehensive comparison of the associations between 15 inflammatory biomarkers and incident lung cancer, and it is the first to investigate the impact of SIRI and AISI on lung cancer development. Third, we stratified analyses by pathological subtypes of lung cancer to examine the relationship between inflammation and each subtype. Fourth, we further adjusted for coexisting inflammatory variables as covariates to ensure our results were not confounded by other inflammatory markers. Fifth, the robustness of our findings was validated through stratified and sensitivity analyses.

However, we acknowledge the following limitations. First, although our new model incorporated additional blood biochemical indicators, several tumor markers (e.g., CEA and CYFRA21-1) were not included due to lack of testing. Second, the analysis may have overlooked certain unmeasured confounders. Third, the Kailuan Study lacks ethnic, gender, and occupational diversity. The cohort predominantly consists of male industrial workers from a single company-based community in northern China, which limits the generalizability of our findings to women, other occupational groups, and populations with different ethnic backgrounds. Furthermore, the other pathological subtype category was heterogeneous and included many unspecified NSCLC cases, which may limit the interpretation of inflammation-related risks for specific rare subtypes. Finally, discrepancies between pathological subtypes and actual diagnoses may exist due to limited availability of historical pathological examinations. Finally, although most primary findings were robust in sensitivity analyses, associations for several markers (e.g., lymphocytes, AISI) were attenuated in cumulative exposure or fully adjusted models, warranting cautious interpretation.

## Conclusion

This large-scale prospective study found that several inflammatory markers, particularly SIRI and AISI, were significantly associated with lung cancer risk, with notable heterogeneity by pathological subtype: significant associations were observed for squamous cell carcinoma but not for adenocarcinoma or small cell lung cancer. SIRI was the strongest independent risk factor, with more pronounced effects in older adults. This is the first report linking SIRI and AISI to incident lung cancer risk, providing subtype-specific evidence for inflammation-based prevention strategies.

## Data Availability

The original contributions presented in the study are included in the article/**Supplementary Material**. Further inquiries can be directed to the corresponding author.

## References

[1] Bray F Laversanne M Sung H Ferlay J Siegel RL Soerjomataram I . Global cancer statistics 2022: GLOBOCAN estimates of incidence and mortality worldwide for 36 cancers in 185 countries. CA Cancer J Clin. (2024) 74:229–63. doi: 10.3322/caac.21834. PMID: 38572751

[2] Wild CP Weiderpass E Stewart BW , editors. (2020). World Cancer Report: Cancer research for cancer prevention. Lyon: International Agency for Research on Cancer. 39432694

[3] Conway EM Pikor LA Kung SHY Hamilton MJ Lam S Lam WL . Macrophages, inflammation, and lung cancer. Am J Respir Crit Care Med. (2016) 193:116–30. doi: 10.1164/rccm.201508-1545ci. PMID: 26583808

[4] Pastorino U Morelli D Leuzzi G Suatoni P Taverna F Bertocchi C . Baseline and postoperative C-reactive protein levels predict mortality in operable lung cancer. Eur J Cancer. (2017) 79:90–7. doi: 10.1016/j.ejca.2017.03.020. PMID: 28472743

[5] Agassandian M Shurin G Ma Y Shurin M . C-reactive protein and lung diseases. Int J Biochem Cell Biol. (2014) 53:77–88. doi: 10.1016/j.biocel.2014.05.016. PMID: 24853773

[6] Wong JYY Bassig BA Loftfield E Hu W Freedman ND Ji BT . White blood cell count and risk of incident lung cancer in the UK Biobank. JNCI Cancer Spectr. (2019) 4:pkz102. doi: 10.1093/jncics/pkz102. PMID: 33313477 PMC7083262

[7] Benej M Capov I Skrickova J Farkasova Ioncikova M Koptikova J Jarkovsky J . Association of the postoperative white blood cells (WBC) count in peripheral blood after radical surgical treatment of left upper lobe non-small cell lung cancer (NSCLC) with overall survival - single center results. Bratisl Lek Listy. (2017) 118:299–301. doi: 10.4149/bll_2017_073. PMID: 28516794

[8] Galvano A Peri M Guarini AA Castiglia M Grassadonia A Rizzo S . Analysis of systemic inflammatory biomarkers in neuroendocrine carcinomas of the lung: prognostic and predictive significance of NLR, LDH, ALI, and LIPI score. Ther Adv Med Oncol. (2020) 12:1758835920942378. doi: 10.1177/1758835920942378. PMID: 32849916 PMC7425322

[9] Mandaliya H Jones M Oldmeadow C Nordman II . Prognostic biomarkers in stage IV non-small cell lung cancer (NSCLC): neutrophil to lymphocyte ratio (NLR), lymphocyte to monocyte ratio (LMR), platelet to lymphocyte ratio (PLR) and advanced lung cancer inflammation index (ALI). Transl Lung Cancer Res. (2019) 8:886–94. doi: 10.21037/tlcr.2019.11.16. PMID: 32010567 PMC6976360

[10] Ruan GT Xie HL Gong YZ Ge YZ Zhang Q Wang ZW . Prognostic importance of systemic inflammation and insulin resistance in patients with cancer: a prospective multicenter study. BMC Cancer. (2022) 22:700. doi: 10.1186/s12885-022-09752-5. PMID: 35752767 PMC9233357

[11] Nagano T Kinoshita F Hashinokuchi A Matsudo K Watanabe K Takamori S . Prognostic impact of C-reactive protein-to-lymphocyte ratio in non-small cell lung cancer: a propensity score-matching analysis. Ann Surg Oncol. (2023) 30:3781–8. doi: 10.1245/s10434-023-13250-8. PMID: 36847957

[12] Hwang J Hur J Eo W An S Kim D Lee S . Clinical significance of C-reactive protein to lymphocyte count ratio as a prognostic factor for survival in non-small cell lung cancer patients undergoing curative surgical resection. J Cancer. (2021) 12:4497–504. doi: 10.7150/jca.58094. PMID: . Published 2021 May 27. 34149913 PMC8210557

[13] Tang C Zhang M Jia H Ma W Xiao H Wang X . The systemic inflammation response index (SIRI) predicts survival in advanced non-small cell lung cancer patients undergoing immunotherapy and the construction of a nomogram model. Front Immunol. (2024) 15:1516737. doi: 10.3389/fimmu.2024.1516737. PMID: 39776905 PMC11703897

[14] Nooh HA Abdellateif MS Refaat L El-Sakhawy YN Bayoumi AM Abdel-Aziz MI . The role of inflammatory indices in the outcome of COVID-19 cancer patients. Med Oncol. (2021) 39:6. doi: 10.1007/s12032-021-01605-8. PMID: 34748094 PMC8573297

[15] Wang F Wu S Song Y Tang X Marshall R Liang M . Waist circumference, body mass index and waist to hip ratio for prediction of the metabolic syndrome in Chinese. Nutr Metab Cardiovasc Dis. (2009) 19:542–7. doi: 10.1016/j.numecd.2008.11.006. PMID: 19188050

[16] Wu S Li Y Jin C Yang P Li D Li H . Intra-individual variability of high-sensitivity C-reactive protein in Chinese general population. Int J Cardiol. (2012) 157:75–9. doi: 10.1016/j.ijcard.2010.12.019. PMID: 21215477

[17] Wu S Huang Z Yang X Zhou Y Wang A Chen L . Prevalence of ideal cardiovascular health and its relationship with the 4-year cardiovascular events in a northern Chinese industrial city. Circ Cardiovasc Qual Outcomes. (2012) 5:487–93. doi: 10.1161/circoutcomes.111.963694. PMID: 22787064

[18] Liu L . 2010 Chinese guidelines for the management of hypertension. Chin J Hypertens. (2011) 19:701–43. doi: 10.1038/ajh.2011.248a. PMID: 22088239

[19] Chinese Diabetes Society . Guidelines for the prevention and control of type 2 diabetes in China (2017 Edition). Chin J Pract Internal Med. (2018) 38:292–344. doi: 10.2337/diacare.27.2007.s133. PMID: 14693949

[20] Nøst TH Alcala K Urbarova I Byrne KS Guida F Sandanger TM . Systemic inflammation markers and cancer incidence in the UK Biobank. Eur J Epidemiol. (2021) 36:841–8. doi: 10.1007/s10654-021-00752-6, PMID: 34036468 PMC8416852

[21] Song M Graubard B Loftfield E Rabkin C Engels E . White blood cell count, neutrophil-to-lymphocyte ratio, and incident cancer in the UK Biobank. Cancer Epidemiol Biomarkers Prev. (2024) 33:821–9. doi: 10.1158/1055-9965.epi-23-1145. PMID: 38568024 PMC11147725

[22] Winther-Larsen A Aggerholm-Pedersen N Sandfeld-Paulsen B . Inflammation scores as prognostic biomarkers in small cell lung cancer: a systematic review and meta-analysis. Syst Rev. (2021) 10:40. doi: 10.1186/s13643-021-01585-w. PMID: 33509254 PMC7844954

[23] Xu Y Zhang L Chen Z Liu H Wang J Li M . The diagnostic value of systemic immune-inflammatory index (SII) and lymphocyte-albumin-neutrophil ratio (LANR) in chronic obstructive pulmonary disease with lung cancer. J Inflammation Res. (2024) 17:5555–65. doi: 10.2147/jir.s474263. PMID: 39185105 PMC11344548

[24] Zhou Y Liu X Wu B Wang Y Chen S Li Z . AGR, LMR and SIRI are the optimal combinations for risk stratification in advanced patients with non-small cell lung cancer following immune checkpoint blockers. Int Immunopharmacol. (2025) 149:114215. doi: 10.1016/j.intimp.2025.114215. PMID: 39904040

[25] Li C Wu J Jiang L Zhang X Wang Y Chen H . The predictive value of inflammatory biomarkers for major pathological response in non-small cell lung cancer patients receiving neoadjuvant chemoimmunotherapy and its association with the immune-related tumor microenvironment: a multi-center study. Cancer Immunol Immunother. (2023) 72:795. doi: 10.1007/s00262-022-03262-w. PMID: 36125538 PMC10992747

[26] Huai Q Luo C Song P . Peripheral blood inflammatory biomarkers dynamics reflect treatment response and predict prognosis in non-small cell lung cancer patients with neoadjuvant immunotherapy. Cancer Sci. (2023) 114:4484–98. doi: 10.1111/cas.15964. PMID: 37731264 PMC10728017

[27] Sheng S Wu Z Zheng H . Prognostic value of C-reactive protein-to-lymphocyte ratio in combined immunotherapy and chemotherapy for small cell lung cancer. J Inflammation Res. (2025) 18:9343–53. doi: 10.2147/jir.s517816. PMID: 40692548 PMC12278946

[28] Muller D Larose T Hodge A . Circulating high sensitivity C reactive protein concentrations and risk of lung cancer: nested case-control study within Lung Cancer Cohort Consortium. BMJ. (2019) 364:k4981. doi: 10.1136/bmj.k4981. PMID: 30606716 PMC6315896

[29] Allin K Bojesen S Nordestgaard B . Inflammatory biomarkers and risk of cancer in 84, 000 individuals from the general population. Int J Cancer. (2016) 139:1493–500. doi: 10.1002/ijc.30194. PMID: 27194008

[30] Qi Q Zhuang L Shen Y . A novel systemic inflammation response index (SIRI) for predicting the survival of patients with pancreatic cancer after chemotherapy. Cancer. (2016) 122:2158–67. doi: 10.1002/cncr.30057. PMID: 27152949

[31] Menyhart O Fekete J Győrffy B . Inflammation and colorectal cancer: a meta-analysis of the prognostic significance of the systemic immune-inflammation index (SII) and the systemic inflammation response index (SIRI). Int J Mol Sci. (2024) 25:8441. doi: 10.3390/ijms25158441. PMID: 39126008 PMC11312822

[32] Zhang S Cheng T . Prognostic and clinicopathological value of systemic inflammation response index (SIRI) in patients with breast cancer: a meta-analysis. Ann Med. (2024) 56:2337729. doi: 10.1080/07853890.2024.2337729. PMID: 38569199 PMC10993763

[33] Wu Z Zhang Z Gu C . Prognostic and clinicopathological impact of systemic inflammation response index (SIRI) on patients with esophageal cancer: a meta-analysis. Syst Rev. (2025) 14:104. doi: 10.1186/s13643-025-02847-7. PMID: 40346701 PMC12063246

