# Supplementary material for: The impact of inflammation on the incidence of different pathological types of lung cancer: the Kailuan study
Source: Front Oncol. 2026 Apr 27;16:1778163. doi: 10.3389/fonc.2026.1778163 (PMC13158107; doi:10.3389/fonc.2026.1778163)
Supplement: Supplementary file 3 [file Table3.docx]

| **Table 1 Baseline characteristics of patients with and without lung cancer** | | | | |
| --- | --- | --- | --- | --- |
| Variables | Total (N=999,25) | Non-lung cancer (N=98,121) | Lung cancer (N=1,804) | P |
| Age, years | 51.85±12.67 | 51.74±12.69 | 57.55±10.31 | <0.001 |
| Women | 20101(20.12) | 19900(20.28) | 201(11.14) | <0.001 |
| Men | 79824(79.88) | 78221(79.72) | 1603(88.86) |  |
| High school or above, n(%) | 19877(19.89) | 19654(20.03) | 223(12.36) | <0.001 |
| Income>1000 yuan/moth, n(%) | 93455(93.53) | 91749(93.51) | 1706(94.57) | 0.069 |
| Current smoking, n(%) | 33487(33.51) | 32726(33.35) | 761(42.18) | <0.001 |
| Family history of cancer, n(%) | 4616(4.62) | 4513(4.60) | 103(5.71) | 0.026 |
| Hypertension, n(%) | 43842(43.87) | 42985(43.81) | 857(47.51) | 0.002 |
| Diabetes, n(%) | 9114(9.12) | 8946(9.12) | 168(9.31) | 0.775 |
| Hs-CRP, mg/L | 0.80(0.30–2.10) | 0.80(0.30–2.10) | 0.95(0.35–2.41) | 0.006 |
| White blood cell, 10^3^/μL | 6.62±1.74 | 6.62±1.74 | 6.80±1.81 | <0.001 |
| Monocytes, 10^3^/μL | 0.42±0.35 | 0.42±0.35 | 0.46±0.61 | 0.001 |
| Lymphocytes, 10^3^/μL | 2.32±0.95 | 2.32±0.95 | 2.37±0.95 | 0.063 |
| Neutrophils, 10^3^/μL | 3.92±1.37 | 3.92±1.37 | 4.05±1.41 | <0.001 |
| Platelet, 10^9^/L | 200.00(168.00–237.00) | 200.00(168.00–237.00) | 198.00(163.00–235.00) | 0.016 |
| HDL-C, mmol/L | 1.55±0.40 | 1.55±0.40 | 1.55±0.41 | 0.772 |
| NLR | 1.86±1.48 | 1.86±1.49 | 1.87±0.86 | 0.626 |
| MLR | 0.17(0.13–0.22) | 0.17(0.13–0.22) | 0.17(0.13–0.23) | 0.471 |
| PLR | 90.42(72.07–113.16) | 90.43(72.11–113.18) | 87.66(68.57–111.18) | 0.507 |
| CLR | 0.36(0.14–1.01) | 0.36(0.13–1.01) | 0.42(0.16–1.23) | <0.001 |
| SII | 335.19(247.68–451.86) | 335.16(247.78–451.56) | 336.67(242.53–464.88) | 0.999 |
| SIRI | 0.62(0.41–0.92) | 0.62(0.41–0.92) | 0.65(0.43–0.97) | 0.007 |
| AISI | 152.37±117.68 | 152.24±117.53 | 158.91±125.13 | 0.021 |
| MHR | 0.24(0.17–0.35) | 0.24(0.17–0.35) | 0.25(0.17–0.36) | 0.367 |
| LHR | 1.66±2.76 | 1.66±2.78 | 1.65±1.08 | 0.857 |
| NHR | 2.79±2.98 | 2.79±3.00 | 2.81±1.39 | 0.754 |
| Abbreviations: NLR, neutrophil to lymphocyte ratio; MLR, monocyte to lymphocyte ratio; PLR, platelet to lymphocyte ratio; CLR, hs-CRP to lymphocyte ratio; SII, neutrophil*platelet/lymphocyte; SIRI, neutrophil*monocyte/lymphocyte; AISI, neutrophil*monocyte*platelet/lymphocyte; MHR, monocyte/high-density lipoprotein cholesterol; LHR, lymphocyte/high-density lipoprotein cholesterol; NHR, neutrophil/high-density lipoprotein cholesterol.  The participants’ baseline characteristics in total and by the incident of lung cancer were presented as mean±standard deviation (SD) and median with interquartile range (IQR) for normally and non-normally distributed continuous variables, respectively, and as numbers with percentages for categorical variables. The distinctions in attributes among groups were scrutinized using the Chi-squared (χ^2^) test or Fisher’s Exact Test for categorical variables, and the Kruskal-Wallis test for continuous variables, respectively. | | | | |

| Table 2 The HR(95%CI) between inflammation and the incidence risk of lung cancer. | | | | | |
| --- | --- | --- | --- | --- | --- |
| Variables | Lung cancer | Lung squamous cell carcinomas | Lung adenocarcinoma | Small cell lung cancer | Other pathological sub-type of lung cancer |
| Hs-CRP | 1.019(0.974-1.066) | 1.125(0.991-1.276) | 0.907(0.803-1.026) | 0.926(0.769-1.114) | 1.034(0.980-1.090) |
| WBC | **1.106(1.057-1.157)** | 1.094(0.952-1.257) | 1.084(0.974-1.206) | 1.071(0.912-1.257) | **1.116(1.057-1.178)** |
| Monocytes | **1.058(1.031-1.086)** | **1.102(1.062-1.143)** | 1.016(0.926-1.115) | 1.045(0.998-1.094) | **1.031(1.001-1.061)** |
| Lymphocytes | **1.046(1.010-1.084)** | 1.036(0.959-1.119) | 0.996(0.914-1.085) | 1.074(0.980-1.178) | **1.055(1.022-1.089)** |
| Neutrophils | **1.081(1.035-1.129)** | 1.067(0.941-1.209) | 1.077(0.980-1.184) | 1.064(0.915-1.238) | **1.083(1.028-1.141)** |
| NLR | 0.996(0.940-1.055) | 1.012(0.956-1.070) | 0.992(0.902-1.092) | 0.947(0.755-1.190) | 0.993(0.940-1.051) |
| MLR | 1.012(0.985-1.038) | **1.026(1.014-1.038)** | 1.002(0.962-1.043) | 0.946(0.791-1.131) | 1.010(0.995-1.026) |
| PLR | 0.992(0.909-1.083) | 0.929(0.590-1.462) | 1.006(0.995-1.017) | 0.999(0.893-1.117) | 0.933(0.741-1.174) |
| CLR | **1.030(1.005-1.057)** | **1.063(1.035-1.092)** | 1.027(0.957-1.102) | **0.668(0.450-0.992)** | 1.017(0.991-1.043) |
| SII | 1.004(0.973-1.036) | 1.009(0.977-1.042) | 1.004(0.990-1.020) | 0.992(0.835-1.178) | 1.004(0.991-1.017) |
| SIRI | **1.535(1.179-1.997)** | **1.773(1.345-2.337)** | 0.522(0.090-3.030) | 0.966(0.428-2.179) | **1.638(1.262-2.129)** |
| AISI | **1.068(1.022-1.117)** | **1.147(1.013-1.299)** | 1.062(0.956-1.179) | 1.092(0.935-1.277) | 1.047(0.990-1.108) |
| MHR | 1.013(0.991-1.035) | **1.027(1.015-1.039)** | 0.900(0.685-1.184) | 0.953(0.846-1.074) | **1.014(1.000-1.027)** |
| LHR | 1.002(0.957-1.049) | 0.990(0.912-1.074) | 0.882(0.688-1.131) | 1.012(0.968-1.057) | 1.009(0.992-1.026) |
| NHR | 1.006(0.970-1.044) | 1.004(0.949-1.063) | 0.986(0.912-1.066) | 1.009(0.965-1.054) | 1.008(0.992-1.023) |
| Abbreviations: NLR, neutrophil to lymphocyte ratio; MLR, monocyte to lymphocyte ratio; PLR, platelet to lymphocyte ratio; CLR, hs-CRP to lymphocyte ratio; SII, neutrophil*platelet/lymphocyte; SIRI, neutrophil*monocyte/lymphocyte; AISI, neutrophil*monocyte*platelet/lymphocyte; MHR, monocyte/high-density lipoprotein cholesterol; LHR, lymphocyte/high-density lipoprotein cholesterol; NHR, neutrophil/high-density lipoprotein cholesterol.  Model adjusted for age, sex, current smoking, education level, income level, hypertension, diabetes and family history of cancer. | | | | | |

| Table 3 Stratification analysis: the HR(95%CI) between inflammation and the incidence risk of lung cancer | | | | | | |
| --- | --- | --- | --- | --- | --- | --- |
| Variables | Sex | | Age | | Smoking status | |
|  | Men | Wmen | ≥60 years old | <60 years old | Current smoking | Never smoking |
| Hs-CRP | 1.039(0.992-1.089) | **0.839(0.709-0.993)** | 1.022(0.954-1.094) | **1.077(1.016-1.142)** | 1.039(0.964-1.120) | 1.011(0.956-1.070) |
| WBC | **1.104(1.053-1.157)** | 1.135(0.983-1.310) | 1.076(0.997-1.162) | **1.089(1.029-1.152)** | **1.139(1.065-1.217)** | **1.081(1.017-1.149)** |
| Monocytes | **1.061(1.035-1.088)** | 0.987(0.843-1.156) | **1.072(1.032-1.114)** | 1.039(0.997-1.083) | **1.048(1.009-1.089)** | **1.071(1.031-1.112)** |
| Lymphocytes | **1.049(1.011-1.089)** | 1.023(0.902-1.160) | 1.055(0.995-1.119) | 1.023(0.970-1.078) | **1.068(1.012-1.128)** | 1.031(0.983-1.082) |
| Neutrophils | **1.077(1.028-1.128)** | 1.110(0.987-1.249) | 1.048(0.970-1.132) | **1.085(1.028-1.145)** | **1.087(1.019-1.160)** | **1.078(1.016-1.143)** |
| NLR | 0.975(0.903-1.054) | 1.022(0.971-1.075) | 0.928(0.818-1.052) | 1.023(0.985-1.064) | 0.965(0.852-1.094) | 1.005(0.960-1.052) |
| MLR | 1.013(0.979-1.048) | 1.010(0.967-1.055) | 1.022(0.986-1.059) | 1.004(0.954-1.056) | 1.035(0.896-1.196) | 1.011(0.983-1.039) |
| PLR | 0.939(0.793-1.112) | 1.002(0.963-1.043) | 0.983(0.788-1.225) | 1.002(0.916-1.096) | 0.838(0.627-1.119) | 1.002(0.964-1.041) |
| CLR | **1.031(1.005-1.057)** | 0.982(0.802-1.203) | 1.021(0.977-1.066) | **1.050(1.025-1.075)** | 1.031(0.998-1.065) | 1.032(0.991-1.074) |
| SII | 1.009(0.955-1.066) | 1.002(0.959-1.048) | 0.995(0.915-1.082) | 1.016(0.971-1.063) | 0.995(0.834-1.186) | 1.005(0.976-1.036) |
| SIRI | **1.556(1.133-2.137)** | 1.493(0.923-2.416) | **2.660(1.853-3.819)** | 1.144(0.682-1.920) | 1.546(0.611-3.912) | **1.516(1.154-1.992)** |
| AISI | **1.069(1.020-1.121)** | 1.061(0.914-1.231) | 1.003(0.926-1.087) | **1.084(1.026-1.145)** | 1.067(0.998-1.140) | **1.073(1.010-1.141)** |
| MHR | 1.022(0.989-1.057) | 1.007(0.962-1.054) | **1.084(1.018-1.154)** | 1.002(0.950-1.056) | 1.034(0.901-1.185) | 1.012(0.989-1.035) |
| LHR | 1.003(0.952-1.056) | 1.001(0.903-1.109) | 1.005(0.945-1.069) | 0.988(0.904-1.080) | **1.017(1.017-1.078)** | 0.987(0.906-1.075) |
| NHR | 1.003(0.961-1.047) | 1.037(0.945-1.139) | 1.001(0.943-1.063) | 1.008(0.951-1.068) | 1.026(0.963-1.092) | 0.998(0.942-1.057) |
| Abbreviations: NLR, neutrophil to lymphocyte ratio; MLR, monocyte to lymphocyte ratio; PLR, platelet to lymphocyte ratio; CLR, hs-CRP to lymphocyte ratio; SII, neutrophil*platelet/lymphocyte; SIRI, neutrophil*monocyte/lymphocyte; AISI, neutrophil*monocyte*platelet/lymphocyte; MHR, monocyte/high-density lipoprotein cholesterol; LHR, lymphocyte/high-density lipoprotein cholesterol; NHR, neutrophil/high-density lipoprotein cholesterol.  Model adjusted for age (exclude age stratified analysis), sex (exclude sex stratified analysis), current smoking (exclude smoking stratified analysis), education level, income level, hypertension, diabetes and family history of cancer. | | | | | | |

| Table 4 Stratification analysis: the HR(95%CI) between inflammation and the incidence risk of lung squamous cell carcinomas | | | | | | |
| --- | --- | --- | --- | --- | --- | --- |
| Variables | Sex | | Age | | Smoking status | |
|  | Men | Wmen | ≥60 years old | <60 years old | Current smoking | Never smoking |
| Hs-CRP | 1.133(0.998-1.286) | 0.740(0.401-1.368) | 0.949(0.728-1.239) | **1.227(1.076-1.398)** | 1.046(0.856-1.278) | **1.190(1.010-1.401)** |
| WBC | 1.076(0.935-1.238) | **1.840(1.073-3.155)** | 1.061(0.763-1.476) | 1.076(0.919-1.260) | 1.145(0.958-1.369) | 1.024(0.819-1.281) |
| Monocytes | **1.101(1.060-1.143)** | **1.182(1.100-1.270)** | **1.150(1.129-1.172)** | **1.061(1.021-1.103)** | **1.081(1.039-1.126)** | **1.144(1.116-1.173)** |
| Lymphocytes | 1.021(0.932-1.117) | **1.196(1.126-1.271)** | 1.017(0.811-1.275) | 1.022(0.922-1.132) | **1.079(1.001-1.163)** | 0.961(0.789-1.170) |
| Neutrophils | 1.063(0.936-1.209) | 1.159(0.781-1.721) | 1.015(0.739-1.394) | 1.071(0.932-1.230) | 1.087(0.948-1.247) | 1.040(0.830-1.303) |
| NLR | 1.016(0.979-1.055) | 0.288(0.015-5.353) | 0.944(0.644-1.383) | 1.033(0.985-1.082) | 0.947(0.706-1.269) | 1.020(0.997-1.044) |
| MLR | **1.030(1.014-1.045)** | 0.991(0.812-1.208) | **1.036(1.011-1.062)** | **1.021(1.004-1.038)** | 1.078(0.990-1.174) | **1.026(1.012-1.039)** |
| PLR | 0.955(0.627-1.454) | 0.321(0.006-17.994) | 1.004(0.980-1.030) | 0.899(0.508-1.593) | 0.747(0.322-1.735) | 1.006(0.978-1.036) |
| CLR | **1.064(1.036-1.093)** | 0.267(0.037-1.931) | 1.053(0.981-1.130) | **1.069(1.037-1.101)** | **1.056(1.026-1.086)** | **1.092(1.036-1.151)** |
| SII | 1.016(0.970-1.064) | 0.775(0.034-17.857) | 0.972(0.555-1.702) | 1.017(0.974-1.061) | 0.935(0.625-1.400) | 1.014(0.996-1.032) |
| SIRI | **1.798(1.350-2.397)** | 1.258(0.624-2.535) | 3.569(2.053-6.206) | 1.292(0.966-1.728) | **2.179(1.312-3.620)** | **1.651(1.169-2.332)** |
| AISI | **1.139(1.001-1.294)** | **1.581(1.129-2.214)** | 1.015(0.806-1.278) | **1.167(1.011-1.347)** | 1.113(0.965-1.283) | 1.194(0.971-1.470) |
| MHR | **1.038(1.016-1.061)** | **1.014(1.005-1.024)** | **1.131(1.046-1.223)** | **1.023(1.008-1.038)** | 1.076(0.999-1.159) | **1.027(1.014-1.040)** |
| LHR | 0.965(0.814-1.144) | **1.033(1.016-1.050)** | 1.013(0.937-1.095) | 0.965(0.797-1.170) | 1.010(0.948-1.077) | 0.919(0.636-1.327) |
| NHR | 0.996(0.915-1.085) | **1.075(1.016-1.137)** | 0.983(0.761-1.271) | 1.009(0.939-1.084) | 1.015(0.906-1.138) | 0.999(0.922-1.082) |
| Abbreviations: NLR, neutrophil to lymphocyte ratio; MLR, monocyte to lymphocyte ratio; PLR, platelet to lymphocyte ratio; CLR, hs-CRP to lymphocyte ratio; SII, neutrophil*platelet/lymphocyte; SIRI, neutrophil*monocyte/lymphocyte; AISI, neutrophil*monocyte*platelet/lymphocyte; MHR, monocyte/high-density lipoprotein cholesterol; LHR, lymphocyte/high-density lipoprotein cholesterol; NHR, neutrophil/high-density lipoprotein cholesterol.  Model adjusted for age (exclude age stratified analysis), sex (exclude sex stratified analysis), current smoking (exclude smoking stratified analysis), education level, income level, hypertension, diabetes and family history of cancer. | | | | | | |
