# Supplementary material for: The impact of inflammation on the incidence of different pathological types of lung cancer: the Kailuan study
Source: Front Oncol. 2026 Apr 27;16:1778163. doi: 10.3389/fonc.2026.1778163 (PMC13158107; doi:10.3389/fonc.2026.1778163)
Supplement: Supplementary file 5 [file Table5.docx]

| Supplementary Table 1. Baseline characteristics of study participants, stratified by the pathological sub-type of incident lung cancer. | | | | | |
| --- | --- | --- | --- | --- | --- |
| Variables | Lung squamous cell carcinomas  (N=215) | Lung adenocarcinoma  (N=317) | Small cell lung cancer  (N=138) | Other pathological types of lung cancer  (N=1134) | P |
| Age, years | 54.01±8.82 | 52.49±8.20 | 53.49±8.36 | 60.13±10.45 | <0.001 |
| Women | 5(2.33) | 71(22.40) | 4(2.90) | 121(10.67) | <0.001 |
| Men | 210(97.67) | 246(77.60) | 134(97.10) | 1013(89.33) |  |
| High school or above, n(%) | 24(11.16) | 48(15.14) | 15(10.87) | 136(11.99) | <0.001 |
| Income>1000 yuan/moth, n(%) | 12(5.58) | 15(4.73) | 3(2.17) | 68(6.00) | 0.160 |
| Smoking status, n(%) |  |  |  |  | <0.001 |
| Never smoking | 93(43.26) | 178(56.15) | 73(52.90) | 582(51.32) |  |
| Former smoking | 8(3.72) | 17(5.36) | 3(2.17) | 89(7.85) |  |
| Occasional smoking | 4(1.86) | 5(1.58) | 4(2.90) | 17(1.50) |  |
| Daily smoking | 110(51.16) | 117(36.91) | 58(42.03) | 446(39.33) |  |
| Family history of cancer, n(%) | 16(7.44) | 25(7.89) | 9(6.52) | 53(4.67) | 0.012 |
| Hypertension, n(%) | 91(42.33) | 126(39.75) | 70(50.72) | 570(50.26) | <0.001 |
| Diabetes, n(%) | 14(6.51) | 24(7.57) | 16(11.59) | 114(10.05) | 0.299 |
| Body mass index, kg/m^2^ | 25.04±3.42 | 24.86±3.48 | 25.17±3.08 | 24.55±3.40 | <0.001 |
| Hs-CRP, mg/L | 0.80(0.32-2.24) | 0.80(0.31-1.75) | 0.88(0.31-1.81) | 1.00(0.38-2.75) | <0.001 |
| White blood cell, 10^3^/μL | 6.89±1.98 | 6.74±1.81 | 6.85±1.75 | 6.80±1.78 | <0.001 |
| Monocytes, 10^3^/μL | 0.61±1.53 | 0.43±0.38 | 0.46±0.27 | 0.43±0.29 | <0.001 |
| Lymphocytes, 10^3^/μL | 2.39±0.80 | 2.32±0.71 | 2.45±1.33 | 2.36±0.98 | 0.292 |
| Neutrophils, 10^3^/μL | 4.08±1.46 | 4.01±1.38 | 4.08±1.37 | 4.05±1.41 | 0.007 |
| Platelet, 10^9^/L | 202.78±56.12 | 216.49±146.09 | 202.53±61.02 | 203.74±97.90 | 0.032 |
| HDL-C, mmol/L | 1.57±0.44 | 1.55±0.37 | 1.54±0.39 | 1.55±0.41 | 0.927 |
| NLR | 1.87±0.92 | 1.83±0.73 | 1.84±0.69 | 1.89±0.90 | 0.948 |
| MLR | 0.18(0.14-0.24) | 0.16(0.12-0.22) | 0.18(0.13-0.24) | 0.17(0.13-0.23) | 0.844 |
| PLR | 86.80(67.50-107.08) | 90.71(73.05-112.83) | 89.35(66.67-115.36) | 86.92(68.40-110.87) | 0.904 |
| CLR | 0.35(0.14-1.32) | 0.36(0.13-0.94) | 0.32(0.16-0.80) | 0.48(0.17-1.40) | <0.001 |
| SII | 326.67(239.55-456.00) | 338.76(253.65-459.36) | 341.14(235.38-483.60) | 339.04(240.11-464.70) | 1.000 |
| SIRI | 0.67(0.49-1.08) | 0.62(0.42-0.94) | 0.70(0.46-0.97) | 0.65(0.42-0.95) | 0.020 |
| AISI | 174.16±148.02 | 157.72±121.48 | 166.68±132.61 | 155.36±120.25 | 0.037 |
| MHR | 0.05-30.54 | 0.04-4.67 | 0.06-1.40 | 0.00-59.28 | 0.580 |
| LHR | 1.66±0.82 | 1.58±0.62 | 1.71±1.08 | 1.66±1.21 | 0.989 |
| NHR | 2.85±1.46 | 2.74±1.16 | 2.88±1.39 | 2.81±1.43 | 0.984 |
| Abbreviations: NLR, neutrophil to lymphocyte ratio; MLR, monocyte to lymphocyte ratio; PLR, platelet to lymphocyte ratio; CLR, hs-CRP to lymphocyte ratio; SII, neutrophil*platelet/lymphocyte; SIRI, neutrophil*monocyte/lymphocyte; AISI, neutrophil*monocyte*platelet/lymphocyte; MHR, monocyte/high-density lipoprotein cholesterol; LHR, lymphocyte/high-density lipoprotein cholesterol; NHR, neutrophil/high-density lipoprotein cholesterol.  The participants’ baseline characteristics in total and by the incident of different pathological lung cancer were presented as mean±standard deviation (SD) and median with interquartile range (IQR) for normally and non-normally distributed continuous variables, respectively, and as numbers with percentages for categorical variables. The distinctions in attributes among groups were scrutinized using the Chi-squared (χ^2^) test or Fisher’s Exact Test for categorical variables, and the Kruskal-Wallis test for continuous variables, respectively. | | | | | |

| Supplementary Table 2 Multiple test correction results (Benjamini-Hochberg FDR) | | | | | | | | | | | |
| --- | --- | --- | --- | --- | --- | --- | --- | --- | --- | --- | --- |
| **Biomarker** | **Time Frame** | Lung Cancer | | Lung Squamous Cell Carcinomas | | Lung Adenocarcinoma | | Small Cell Lung Cancer | | Other Pathological Sub-type of Lung Cancer | |
|  |  | **Raw P** | **FDR P** | **Raw P** | **FDR P** | **Raw P** | **FDR P** | **Raw P** | **FDR P** | **Raw P** | **FDR P** |
| Hs-CRP | long-term | 0.6384 | 0.8495 | 0.1031 | 0.2209 | 0.1029 | 0.6465 | 0.3503 | 0.8051 | 0.1201 | 0.2252 |
| Hs-CRP | 10-year | 0.2484 | 0.4140 | 0.0718 | 0.1539 | 0.1327 | 0.6635 | 0.4075 | 0.7946 | 0.1077 | 0.2019 |
| WBC | long-term | 0.0001 | 0.0015 | 0.2123 | 0.3981 | 0.1293 | 0.6465 | 0.3736 | 0.8051 | 0.0001 | 0.0010 |
| WBC | 10-year | 0.0001 | 0.0015 | 0.2225 | 0.4172 | 0.1324 | 0.6635 | 0.3772 | 0.7946 | 0.0001 | 0.0015 |
| Monocytes | long-term | 0.0001 | 0.0008 | 0.0001 | 0.0015 | 0.7524 | 0.9405 | 0.0531 | 0.3983 | 0.0113 | 0.0339 |
| Monocytes | 10-year | 0.0001 | 0.0008 | 0.0001 | 0.0015 | 0.7134 | 0.9260 | 0.0580 | 0.4350 | 0.0147 | 0.0441 |
| Lymphocytes | long-term | 0.0612 | 0.1311 | 0.3288 | 0.4994 | 0.9694 | 0.9694 | 0.1098 | 0.5490 | 0.0002 | 0.0015 |
| Lymphocytes | 10-year | 0.0064 | 0.0160 | 0.3996 | 0.5994 | 0.9536 | 0.9536 | 0.1233 | 0.6165 | 0.0003 | 0.0015 |
| Neutrophils | long-term | 0.0024 | 0.0090 | 0.3329 | 0.4994 | 0.1242 | 0.6465 | 0.4125 | 0.8051 | 0.0020 | 0.0075 |
| Neutrophils | 10-year | 0.0003 | 0.0015 | 0.3264 | 0.5440 | 0.1197 | 0.6635 | 0.4080 | 0.7946 | 0.0017 | 0.0064 |
| NLR | long-term | 0.9428 | 0.9505 | 0.7772 | 0.8406 | 0.8196 | 0.9457 | 0.6125 | 0.9012 | 0.7200 | 0.7200 |
| NLR | 10-year | 0.8208 | 0.8470 | 0.6416 | 0.8020 | 0.8703 | 0.9536 | 0.6434 | 0.8655 | 0.7389 | 0.7389 |
| MLR | long-term | 0.1681 | 0.2802 | 0.0001 | 0.0008 | 0.9272 | 0.9694 | 0.5534 | 0.9012 | 0.1808 | 0.2465 |
| MLR | 10-year | 0.3910 | 0.5865 | 0.0001 | 0.0008 | 0.9246 | 0.9536 | 0.5421 | 0.8655 | 0.1988 | 0.2485 |
| PLR | long-term | 0.8540 | 0.9505 | 0.6906 | 0.8406 | 0.2586 | 0.8082 | 0.9575 | 0.9575 | 0.4211 | 0.4859 |
| PLR | 10-year | 0.7868 | 0.8470 | 0.7559 | 0.8413 | 0.3137 | 0.7898 | 0.9875 | 0.9875 | 0.4267 | 0.4923 |
| CLR | long-term | 0.0060 | 0.0150 | 0.0001 | 0.0005 | 0.4887 | 0.8082 | 0.0402 | 0.3983 | 0.1546 | 0.2465 |
| CLR | 10-year | 0.0166 | 0.0356 | 0.0001 | 0.0005 | 0.4373 | 0.7898 | 0.0446 | 0.4350 | 0.1719 | 0.2344 |
| SII | long-term | 0.5829 | 0.8495 | 0.7206 | 0.8406 | 0.5388 | 0.8082 | 0.9045 | 0.9575 | 0.6794 | 0.7200 |
| SII | 10-year | 0.8078 | 0.8470 | 0.5882 | 0.8020 | 0.5656 | 0.8484 | 0.9317 | 0.9875 | 0.6589 | 0.7060 |
| SIRI | long-term | 0.0001 | 0.0005 | 0.0001 | 0.0004 | 0.4771 | 0.8082 | 0.9481 | 0.9575 | 0.0002 | 0.0010 |
| SIRI | 10-year | 0.0013 | 0.0049 | 0.0001 | 0.0004 | 0.4739 | 0.7898 | 0.9363 | 0.9875 | 0.0002 | 0.0015 |
| AISI | long-term | 0.0034 | 0.0102 | 0.0363 | 0.0908 | 0.3114 | 0.8082 | 0.2707 | 0.8051 | 0.0879 | 0.1884 |
| AISI | 10-year | 0.0030 | 0.0090 | 0.0322 | 0.0805 | 0.2617 | 0.7898 | 0.2576 | 0.7946 | 0.0875 | 0.1875 |
| MHR | long-term | 0.0761 | 0.1427 | 0.0001 | 0.0003 | 0.4721 | 0.8082 | 0.4294 | 0.8051 | 0.0339 | 0.0848 |
| MHR | 10-year | 0.2184 | 0.4095 | 0.0001 | 0.0003 | 0.4495 | 0.7898 | 0.4238 | 0.7946 | 0.0371 | 0.0928 |
| LHR | long-term | 0.9505 | 0.9505 | 0.7846 | 0.8406 | 0.3456 | 0.8082 | 0.6609 | 0.9012 | 0.1807 | 0.2465 |
| LHR | 10-year | 0.8470 | 0.8470 | 0.7852 | 0.8413 | 0.3411 | 0.7898 | 0.6063 | 0.8655 | 0.1640 | 0.2344 |
| NHR | long-term | 0.6796 | 0.8495 | 0.9711 | 0.9711 | 0.7150 | 0.9405 | 0.7768 | 0.9575 | 0.2217 | 0.2771 |
| NHR | 10-year | 0.6508 | 0.8470 | 0.9149 | 0.9149 | 0.7408 | 0.9260 | 0.6924 | 0.8655 | 0.1692 | 0.2344 |
| Abbreviations: NLR, neutrophil to lymphocyte ratio; MLR, monocyte to lymphocyte ratio; PLR, platelet to lymphocyte ratio; CLR, hs-CRP to lymphocyte ratio; SII, neutrophil*platelet/lymphocyte; SIRI, neutrophil*monocyte/lymphocyte; AISI, neutrophil*monocyte*platelet/lymphocyte; MHR, monocyte/high-density lipoprotein cholesterol; LHR, lymphocyte/high-density lipoprotein cholesterol; NHR, neutrophil/high-density lipoprotein cholesterol. | | | | | | | | | | | |

| Supplementary Table 3 Sensitivity analysis 1: the HR(95%CI) between inflammation and the incidence risk of lung cancer | | | | | |
| --- | --- | --- | --- | --- | --- |
| Variables | Lung cancer | Lung squamous cell carcinomas | Lung adenocarcinoma | Small cell lung cancer | Other pathological Sub-type of lung cancer |
| Hs-CRP | 1.016(0.969-1.065) | 1.131(0.993-1.289) | 0.911(0.803-1.032) | 0.915(0.752-1.112) | 1.028(0.972-1.087) |
| WBC | **1.110(1.060-1.163)** | 1.066(0.924-1.229) | 1.086(0.973-1.213) | 1.057(0.894-1.249) | **1.128(1.067-1.192)** |
| Monocytes | **1.061(1.034-1.088)** | **1.102(1.059-1.147)** | 1.011(0.910-1.124) | 1.042(0.990-1.097) | **1.035(1.008-1.063)** |
| Lymphocytes | **1.053(1.016-1.090)** | 1.041(0.966-1.122) | 0.993(0.907-1.087) | 1.070(0.967-1.183) | **1.063(1.032-1.096)** |
| Neutrophils | **1.080(1.033-1.129)** | 1.031(0.902-1.178) | 1.080(0.982-1.188) | 1.057(0.901-1.239) | **1.088(1.032-1.147)** |
| NLR | 0.987(0.921-1.058) | 0.962(0.784-1.179) | 0.997(0.915-1.086) | 0.921(0.719-1.181) | 0.991(0.929-1.057) |
| MLR | 1.012(0.986-1.039) | **1.027(1.014-1.039)** | 1.002(0.960-1.045) | 0.914(0.693-1.204) | 1.011(0.996-1.027) |
| PLR | 0.964(0.827-1.122) | 0.821(0.487-1.384) | 1.006(0.994-1.018) | 1.003(0.942-1.067) | 0.881(0.671-1.156) |
| CLR | 1.009(0.970-1.049) | **1.043(1.009-1.077)** | 1.031(0.969-1.098) | **0.625(0.401-0.974)** | 0.999(0.963-1.036) |
| SII | 1.003(0.964-1.043) | 0.937(0.682-1.289) | 1.005(0.990-1.020) | 0.994(0.854-1.157) | 1.003(0.986-1.020) |
| SIRI | **1.538(1.178-2.008)** | **1.766(1.310-2.380)** | 0.482(0.065-3.596) | 0.821(0.218-3.086) | **1.657(1.276-2.151)** |
| AISI | **1.063(1.014-1.113)** | 1.137(0.997-1.297) | 1.058(0.949-1.180) | 1.079(0.916-1.271) | 1.043(0.983-1.107) |
| MHR | 1.014(0.992-1.036) | **1.027(1.015-1.040)** | 0.896(0.664-1.209) | 0.943(0.818-1.087) | **1.015(1.001-1.028)** |
| LHR | 1.005(0.962-1.049) | 0.976(0.856-1.113) | 0.888(0.692-1.138) | 1.010(0.961-1.062) | 1.012(0.997-1.027) |
| NHR | 1.006(0.968-1.046) | 0.952(0.787-1.152) | 0.992(0.928-1.060) | 1.007(0.957-1.059) | 1.010(0.995-1.024) |
| Sensitivity analysis 1 was excluded participants with a diagnosis of lung cancer less then 1 year.  Abbreviations: NLR, neutrophil to lymphocyte ratio; MLR, monocyte to lymphocyte ratio; PLR, platelet to lymphocyte ratio; CLR, hs-CRP to lymphocyte ratio; SII, neutrophil*platelet/lymphocyte; SIRI, neutrophil*monocyte/lymphocyte; AISI, neutrophil*monocyte*platelet/lymphocyte; MHR, monocyte/high-density lipoprotein cholesterol; LHR, lymphocyte/high-density lipoprotein cholesterol; NHR, neutrophil/high-density lipoprotein cholesterol.  Model adjusted for age , sex , current smoking , education level, income level, hypertension, diabetes, body mass index and family history of cancer. | | | | | |

| Supplementary Table 4 Sensitivity analysis 2: the HR(95%CI) between inflammation and the incidence risk of lung cancer | | | | | |
| --- | --- | --- | --- | --- | --- |
| Variables | Lung cancer | Lung squamous cell carcinomas | Lung adenocarcinoma | Small cell lung cancer | Other pathological Sub-type of lung cancer |
| TWC^#^-hs-CRP | 1.045(0.988-1.105) | 0.948(0.799-1.126) | 0.951(0.828-1.093) | 0.931(0.755-1.149) | **1.099(1.028-1.175)** |
| TWC-WBC | **1.125(1.065-1.189)** | 1.063(0.896-1.261) | 1.037(0.906-1.188) | 1.141(0.888-1.467) | **1.161(1.083-1.244)** |
| TWC-Monocytes | 1.029(0.982-1.078) | **1.068(1.025-1.112)** | 0.949(0.831-1.084) | 0.986(0.842-1.155) | **1.036(1.000-1.073)** |
| TWC-Lymphocytes | **1.050(1.001-1.102)** | 1.083(0.978-1.199) | 1.009(0.901-1.129) | 1.017(0.859-1.204) | **1.060(1.013-1.109)** |
| TWC-Neutrophils | **1.124(1.065-1.187)** | 1.028(0.865-1.222) | 1.026(0.900-1.170) | 1.198(0.931-1.543) | **1.158(1.081-1.240)** |
| TWC-NLR | 1.008(0.955-1.063) | 0.916(0.737-1.137) | 0.938(0.803-1.096) | 1.009(0.840-1.213) | 1.022(0.988-1.056) |
| TWC-MLR | 0.988(0.899-1.085) | 0.992(0.950-1.036) | 0.996(0.909-1.092) | 0.376(0.040-3.564) | 0.991(0.943-1.041) |
| TWC-PLR | 0.999(0.940-1.061) | **1.018(1.000-1.037)** | 0.958(0.708-1.296) | 1.000(0.918-1.089) | 0.953(0.814-1.116) |
| TWC-CLR | **1.037(1.018-1.055)** | 0.971(0.795-1.186) | **0.701(0.543-0.903)** | **0.607(0.373-0.988)** | **1.043(1.024-1.062)** |
| TWC-SII | 1.013(0.990-1.037) | 1.017(0.998-1.036) | 0.936(0.732-1.197) | 1.016(0.998-1.033) | **1.014(1.003-1.026)** |
| TWC-SIRI | 1.027(0.994-1.061) | 1.015(0.969-1.064) | 0.988(0.848-1.151) | 1.019(0.944-1.099) | **1.033(1.009-1.057)** |
| TWC-AISI | **1.107(1.051-1.167)** | **1.156(1.005-1.329)** | 1.009(0.884-1.151) | 1.029(0.872-1.215) | **1.129(1.055-1.208)** |
| TWC-MHR | 1.002(0.948-1.059) | 0.969(0.885-1.061) | 0.867(0.657-1.143) | 0.934(0.770-1.132) | 1.016(0.982-1.050) |
| TWC-LHR | 1.011(0.965-1.060) | 0.911(0.679-1.222) | 0.978(0.882-1.084) | 1.029(0.986-1.074) | **1.022(1.000-1.044)** |
| TWC-NHR | **1.041(1.001-1.083)** | 0.853(0.677-1.076) | 0.993(0.904-1.091) | **1.080(1.034-1.128)** | **1.053(1.028-1.079)** |
| Sensitivity analysis 2 was calculated the cumulative value of each inflammatory marker using three repeated measurements (collected in 2006, 2008, and 2010). | | | | | |
| Abbreviations: ^#^TWC stands for Time-Weighted Cumulative Value. NLR, neutrophil to lymphocyte ratio; MLR, monocyte to lymphocyte ratio; PLR, platelet to lymphocyte ratio; CLR, hs-CRP to lymphocyte ratio; SII, neutrophil*platelet/lymphocyte; SIRI, neutrophil*monocyte/lymphocyte; AISI, neutrophil*monocyte*platelet/lymphocyte; MHR, monocyte/high-density lipoprotein cholesterol; LHR, lymphocyte/high-density lipoprotein cholesterol; NHR, neutrophil/high-density lipoprotein cholesterol. | | | | | |
| Model adjusted for age , sex , current smoking , education level, income level, body mass index, hypertension, diabetes, body mass index and family history of cancer. | | | | | |

| Supplementary Table 5 Sensitivity analysis 3: the HR(95%CI) between inflammation and the incidence risk of lung cancer | | | | |  | |
| --- | --- | --- | --- | --- | --- | --- |
| Variables |  | Lung cancer | Lung squamous cell carcinomas | Lung adenocarcinoma | Small cell lung cancer | Other pathological Sub-type of lung cancer |
| WBC, ×10⁹/L | <4 | Ref | Ref | Ref | Ref | Ref |
|  | 4-10 | **1.318(1.057-1.644)** | 1.618(0.794-3.295) | 1.655(0.929-2.948) | 2.032(0.748-5.518) | 1.163(0.894-1.513) |
|  | ≥10 | **1.975(1.480-2.636)** | **2.932(1.255-6.847)** | **2.251(1.074-4.715)** | 2.979(0.900-9.866) | **1.685(1.179-2.408)** |
| Monocytes, ×10⁹/L | <0.1 | Ref | Ref | Ref | Ref | Ref |
|  | 0.1-0.6 | **1.364(1.117-1.665)** | 1.597(0.865-2.947) | **1.814(1.073-3.066)** | 1.876(0.831-4.237) | 1.196(0.941-1.520) |
|  | ≥0.6 | **1.638(1.319-2.035)** | **2.408(1.258-4.608)** | **2.003(1.139-3.521)** | **2.363(1.001-5.575)** | **1.381(1.060-1.799)** |
| Lymphocytes, ×10⁹/L | <1.1 | Ref | Ref | Ref | Ref | Ref |
|  | 1.1-3.2 | **1.505(1.213-1.866)** | 1.902(0.975-3.712) | 1.694(0.988-2.905) | 1.320(0.645-2.701) | **1.428(1.092-1.867)** |
|  | ≥3.2 | **1.857(1.453-2.374)** | 2.095(0.988-4.442) | **2.045(1.114-3.756)** | 1.426(0.616-3.301) | **1.837(1.352-2.496)** |
| Neutrophils, ×10⁹/L | <1.8 | Ref | Ref | Ref | Ref | Ref |
|  | 1.8-6.3 | **1.242(1.012-1.526)** | 1.704(0.872-3.329) | **1.835(1.028-3.276)** | 2.983(0.945-9.416) | 1.018(0.803-1.291) |
|  | ≥6.3 | **1.595(1.216-2.093)** | 1.762(0.742-4.186) | **2.265(1.099-4.669)** | **4.864(1.369-17.283)** | 1.316(0.948-1.827) |
| MLR | <0.14 | Ref | Ref | Ref | Ref | Ref |
|  | 0.14-0.20 | 0.966(0.857-1.088) | 1.109(0.771-1.595) | 0.901(0.684-1.187) | 1.052(0.682-1.622) | 0.953(0.820-1.108) |
|  | ≥0.20 | 1.094(0.974-1.228) | **1.521(1.082-2.137)** | 0.966(0.733-1.273) | 1.218(0.801-1.853) | 1.043(0.901-1.208) |
| CLR | <0.19 | Ref | Ref | Ref | Ref | Ref |
|  | 0.19-0.69 | 1.065(0.943-1.202) | 0.935(0.670-1.304) | 1.114(0.845-1.470) | 1.298(0.857-1.964) | 1.048(0.895-1.228) |
|  | ≥0.69 | **1.147(1.016-1.294)** | 1.136(0.807-1.598) | 1.045(0.782-1.396) | 0.907(0.573-1.435) | **1.200(1.028-1.400)** |
| SIRI | <0.48 | Ref | Ref | Ref | Ref | Ref |
|  | 0.48-0.80 | 1.115(0.988-1.257) | 1.425(0.993-2.046) | 1.160(0.877-1.534) | 1.187(0.763-1.847) | 1.041(0.893-1.212) |
|  | ≥0.80 | **1.199(1.065-1.350)** | **1.450(1.015-2.071)** | 1.181(0.888-1.569) | 1.250(0.812-1.925) | 1.147(0.988-1.332) |
| AISI | <90.12 | Ref | Ref | Ref | Ref | Ref |
|  | 90.12-161.96 | 1.012(0.898-1.140) | 1.358(0.951-1.939) | 0.960(0.725-1.272) | 1.130(0.726-1.757) | 0.958(0.824-1.114) |
|  | ≥161.96 | **1.149(1.022-1.293)** | 1.346(0.944-1.920) | 1.108(0.839-1.463) | 1.292(0.839-1.991) | 1.108(0.956-1.285) |
| MHR | <0.19 | Ref | Ref | Ref | Ref | Ref |
|  | 0.19-0.31 | **1.195(1.060-1.346)** | 1.159(0.812-1.654) | 0.960(0.723-1.274) | 1.115(0.710-1.752) | **1.281(1.102-1.490)** |
|  | ≥0.31 | **1.253(1.110-1.414)** | 1.266(0.895-1.791) | 1.066(0.798-1.425) | 1.404(0.905-2.180) | **1.275(1.091-1.491)** |
| Sensitivity analysis 3 refers to the grouping of inflammatory indicators. | | | | | | |
| Abbreviations: WBC, white blood cell; MLR, monocyte to lymphocyte ratio; PLR, platelet to lymphocyte ratio; CLR, hs-CRP to lymphocyte ratio; SIRI, neutrophil*monocyte/lymphocyte; AISI, neutrophil*monocyte*platelet/lymphocyte; MHR, monocyte/high-density lipoprotein cholesterol. | | | | | | |
| Model adjusted for age , sex , current smoking , education level, income level, body mass index, hypertension, diabetes, body mass index and family history of cancer. | | | | | | |

| Supplementary Table 6 Sensitivity analysis 4: The HR(95%CI) between inflammation and the incidence risk of lung cancer further adjusted for hs-CRP and WBC. | | | | | |
| --- | --- | --- | --- | --- | --- |
| Variables | Lung cancer | Lung squamous cell carcinomas | Lung adenocarcinoma | Small cell lung cancer | Other pathological Sub-type of lung cancer |
| Hs-CRP | 1.025(0.979-1.072) | 1.128(0.994-1.281) | 0.910(0.806-1.028) | 0.900(0.742-1.092) | 1.042(0.989-1.099) |
| WBC | **1.097(1.047-1.150)** | 1.044(0.907-1.201) | 1.095(0.978-1.226) | 1.103(0.935-1.301) | **1.104(1.044-1.168)** |
| Monocytes | **1.053(1.023-1.085)** | **1.102(1.061-1.145)** | 0.995(0.864-1.146) | 1.041(0.988-1.097) | 1.016(0.977-1.057) |
| Lymphocytes | 1.018(0.971-1.068) | 1.007(0.915-1.109) | 0.932(0.812-1.071) | 1.066(0.949-1.198) | 1.029(0.980-1.080) |
| Neutrophils | 0.962(0.877-1.055) | 0.867(0.666-1.130) | 1.028(0.856-1.235) | 0.986(0.717-1.355) | 0.959(0.856-1.075) |
| NLR | 0.952(0.877-1.033) | 0.934(0.733-1.190) | 0.956(0.819-1.116) | 0.942(0.735-1.207) | 0.953(0.863-1.052) |
| MLR | 1.011(0.984-1.039) | **1.026(1.013-1.038)** | 1.001(0.958-1.046) | 0.950(0.806-1.120) | 1.010(0.994-1.026) |
| PLR | 1.003(0.924-1.089) | 0.864(0.537-1.391) | **1.029(1.006-1.052)** | 1.009(0.947-1.076) | 0.950(0.752-1.199) |
| CLR | 0.817(0.614-1.088) | 0.894(0.411-1.943) | 1.006(0.592-1.710) | 0.495(0.131-1.876) | 0.786(0.556-1.111) |
| SII | 0.969(0.861-1.092) | 0.827(0.528-1.296) | 1.003(0.906-1.109) | 0.955(0.673-1.356) | 0.974(0.857-1.106) |
| SIRI | **1.454(1.081-1.956)** | **1.711(1.230-2.381)** | 0.098(0.002-5.774) | 0.843(0.287-2.473) | **1.570(1.153-2.137)** |
| AISI | 1.021(0.963-1.082) | 1.093(0.939-1.271) | 1.005(0.875-1.156) | 1.088(0.879-1.345) | 1.000(0.929-1.077) |
| MHR | 1.013(0.991-1.036) | **1.026(1.014-1.038)** | 0.823(0.393-1.723) | 0.942(0.822-1.081) | **1.014(1.000-1.028)** |
| LHR | 0.995(0.938-1.055) | 0.983(0.884-1.094) | 0.750(0.507-1.111) | 1.008(0.952-1.067) | 1.005(0.983-1.028) |
| NHR | 0.982(0.911-1.059) | 0.951(0.751-1.204) | 0.888(0.706-1.117) | 0.996(0.918-1.081) | 0.994(0.960-1.029) |
| Abbreviations: NLR, neutrophil to lymphocyte ratio; MLR, monocyte to lymphocyte ratio; PLR, platelet to lymphocyte ratio; CLR, hs-CRP to lymphocyte ratio; SII, neutrophil*platelet/lymphocyte; SIRI, neutrophil*monocyte/lymphocyte; AISI, neutrophil*monocyte*platelet/lymphocyte; MHR, monocyte/high-density lipoprotein cholesterol; LHR, lymphocyte/high-density lipoprotein cholesterol; NHR, neutrophil/high-density lipoprotein cholesterol.  Model adjusted for age, sex, current smoking, education level, income level, hypertension, diabetes, body mass index, family history of cancer, hs-CRP(No adjustment was made when hs-CRP was used as an independent variable) and WBC(No adjustment was made when WBC was used as an independent variable) . | | | | | |

| Supplementary Table 7 Stratification analysis: the HR(95%CI) between inflammation and the incidence risk of lung adenocarcinoma | | | | | | | | |
| --- | --- | --- | --- | --- | --- | --- | --- | --- |
| Variables | Sex | | Age | | Smoking status | | | |
|  | Men | Wmen | ≥60 years old | <60 years old | Never smoking | Former smoking | Occasional smoking | Daily smoking |
| Hs-CRP | 0.930(0.816-1.060) | 0.837(0.616-1.136) | 0.907(0.681-1.210) | 0.919(0.803-1.050) | 0.922(0.794-1.070) | 0.533(0.314-0.903) | 1.327(0.640-2.752) | 0.913(0.733-1.138) |
| WBC | **1.132(1.011-1.266)** | 0.884(0.661-1.182) | 1.091(0.852-1.398) | 1.094(0.972-1.232) | 1.053(0.895-1.237) | 0.951(0.635-1.424) | 0.943(0.623-1.428) | 1.148(0.982-1.342) |
| Monocytes | 1.044(0.989-1.101) | 0.648(0.421-0.996) | **1.054(1.000-1.110)** | 1.008(0.886-1.148) | 0.981(0.869-1.108) | 0.683(0.296-1.578) | 0.570(0.321-1.013) | 1.042(0.973-1.116) |
| Lymphocytes | 1.021(0.946-1.102) | 0.842(0.593-1.197) | 1.065(0.924-1.227) | 0.980(0.882-1.089) | 0.899(0.750-1.077) | 1.091(0.923-1.289) | 0.844(0.492-1.449) | 1.072(0.998-1.152) |
| Neutrophils | 1.103(0.996-1.222) | 0.966(0.754-1.238) | 1.031(0.822-1.293) | 1.092(0.992-1.201) | 1.090(0.955-1.243) | 0.871(0.582-1.305) | 1.036(0.740-1.451) | 1.080(0.941-1.240) |
| NLR | 0.993(0.874-1.130) | 0.993(0.880-1.119) | 0.790(0.526-1.186) | 1.014(0.977-1.053) | 1.015(0.991-1.040) | 0.493(0.226-1.075) | 0.889(0.488-1.617) | 0.905(0.657-1.247) |
| MLR | 1.013(0.981-1.046) | 0.039(0.001-2.823) | 0.974(0.878-1.079) | 1.003(0.966-1.042) | 0.820(0.564-1.193) | 0.001(0.000-8.131) | - | 1.104(0.993-1.228) |
| PLR | 0.998(0.865-1.151) | 1.006(0.995-1.017) | 1.000(0.986-1.014) | 1.021(0.994-1.049) | 1.007(0.997-1.017) | 1.076(0.628-1.845) | 1.463(0.063-33.956) | 0.823(0.341-1.985) |
| CLR | 1.032(0.971-1.097) | 0.936(0.657-1.333) | 0.977(0.730-1.308) | 1.040(0.986-1.097) | 1.043(0.919-1.184) | 0.174(0.044-0.683) | 1.005(0.901-1.122) | 1.039(0.970-1.112) |
| SII | 1.015(0.976-1.055) | 1.002(0.981-1.023) | 0.994(0.943-1.048) | 1.018(0.989-1.047) | 1.007(0.995-1.019) | 0.780(0.230-2.647) | 1.113(0.438-2.825) | 0.913(0.581-1.436) |
| SIRI | 0.833(0.327-2.121) | - | 1.035(0.179-5.994) | 0.562(0.098-3.233) | 0.825(0.444-1.531) | - | - | - |
| AISI | 1.087(0.972-1.216) | 0.934(0.691-1.264) | 1.204(0.978-1.481) | 1.044(0.926-1.176) | 1.125(0.982-1.289) | 0.912(0.475-1.754) | 0.812(0.571-1.154) | 1.002(0.844-1.190) |
| MHR | 0.959(0.843-1.092) | 0.049(0.002-1.415) | 0.939(0.758-1.162) | 0.900(0.656-1.235) | 0.697(0.273-1.780) | - |  | 1.033(0.883-1.208) |
| LHR | 0.922(0.737-1.155) | 0.747(0.326-1.713) | 0.908(0.548-1.506) | 0.884(0.671-1.163) | 0.713(0.431-1.181) | 0.436(0.057-3.333) | 0.436(0.096-1.975) | 1.007(0.948-1.071) |
| NHR | 0.995(0.933-1.060) | 0.953(0.709-1.282) | 0.823(0.510-1.328) | 1.003(0.952-1.057) | 1.000(0.943-1.061) | 0.328(0.073-1.473) | 0.789(0.515-1.210) | 0.996(0.893-1.112) |
| Abbreviations: NLR, neutrophil to lymphocyte ratio; MLR, monocyte to lymphocyte ratio; PLR, platelet to lymphocyte ratio; CLR, hs-CRP to lymphocyte ratio; SII, neutrophil*platelet/lymphocyte; SIRI, neutrophil*monocyte/lymphocyte; AISI, neutrophil*monocyte*platelet/lymphocyte; MHR, monocyte/high-density lipoprotein cholesterol; LHR, lymphocyte/high-density lipoprotein cholesterol; NHR, neutrophil/high-density lipoprotein cholesterol.  Model adjusted for age (exclude age stratified analysis), sex (exclude sex stratified analysis), current smoking (exclude smoking stratified analysis), education level, income level, hypertension, diabetes, body mass index and family history of cancer.  -: After stratification by sex, age or smoking status the limited number of female or different age stage participants led to issues such as model non-convergence, insufficient sample size, and low event counts, among other issues. Consequently, HR (95% CI) could not be calculated for some variables. | | | | | | | | |

| Supplementary Table 8 Stratification analysis: the HR(95%CI) between inflammation and the incidence risk of small cell lung cancer | | | | | | | | |
| --- | --- | --- | --- | --- | --- | --- | --- | --- |
| Variables | Sex | | Age | | Smoking status | | | |
|  | Men | Women | ≥60 years old | <60 years old | Never smoking | Former smoking | Occasional smoking | Daily smoking |
| Hs-CRP | 0.931(0.770-1.124) | **0.743(0.580-0.951)** | 0.842(0.588-1.207) | 0.952(0.773-1.172) | 0.813(0.601-1.101) | 0.953(0.530-1.712) | 0.886(0.606-1.295) | 1.093(0.887-1.347) |
| WBC | 1.078(0.915-1.270) | 0.903(0.331-2.465) | 0.996(0.690-1.437) | 1.095(0.915-1.311) | 1.089(0.880-1.348) | 1.847(0.838-4.071) | **1.798(1.137-2.841)** | 0.969(0.740-1.270) |
| Monocytes | **1.046(1.001-1.094)** | 0.704(0.114-4.366) | 0.558(0.273-1.141) | **1.062(1.023-1.102)** | **1.068(1.019-1.119)** | 1.109(0.385-3.196) | 1.511(0.774-2.952) | 0.981(0.819-1.175) |
| Lymphocytes | 1.074(0.976-1.182) | 1.023(0.583-1.795) | 1.019(0.850-1.221) | 1.079(0.982-1.186) | 1.091(0.998-1.192) | **1.214(1.055-1.397)** | 2.256(0.807-6.302) | 0.906(0.630-1.302) |
| Neutrophils | 1.069(0.916-1.246) | 0.954(0.407-2.238) | 1.006(0.696-1.455) | 1.080(0.920-1.269) | 1.071(0.877-1.309) | 1.222(0.454-3.288) | **1.497(1.015-2.209)** | 1.006(0.772-1.313) |
| NLR | 0.966(0.783-1.192) | 0.238(0.140-0.406) | 0.717(0.410-1.254) | 1.005(0.859-1.176) | 0.950(0.721-1.252) | 0.106(0.010-1.177) | 1.201(0.243-5.942) | 0.974(0.686-1.382) |
| MLR | 0.957(0.823-1.113) | - | - | 0.994(0.959-1.030) | 0.933(0.749-1.162) | - | - | 0.979(0.776-1.235) |
| PLR | 1.005(0.919-1.099) | 0.623(0.056-6.946) | 0.645(0.219-1.901) | 1.008(0.958-1.061) | 0.912(0.423-1.967) | - | 1.502(0.032-69.775) | 1.109(0.924-1.330) |
| CLR | 0.674(0.451-1.007) | 0.402(0.136-1.183) | 0.534(0.242-1.177) | 0.717(0.460-1.118) | 0.490(0.244-0.981) | 0.153(0.039-0.598) | 0.500(0.173-1.444) | 0.929(0.718-1.201) |
| SII | 1.002(0.901-1.114) | 0.197(0.048-0.818) | 0.764(0.300-1.948) | 1.007(0.948-1.070) | 0.970(0.653-1.440) | 0.315(0.020-4.975) | 1.890(0.960-3.720) | 1.006(0.701-1.443) |
| SIRI | 1.013(0.494-2.076) | - | - | 1.232(0.862-1.761) | 0.876(0.350-2.191) | - | - | 1.197(0.214-6.698) |
| AISI | 1.101(0.942-1.286) | 0.594(0.126-2.802) | 0.710(0.451-1.120) | 1.160(0.989-1.361) | 1.084(0.852-1.380) | 0.996(0.358-2.768) | 1.564(0.998-2.449) | 1.085(0.868-1.355) |
| MHR | 0.959(0.861-1.068) | - | - | 0.988(0.936-1.044) | 0.974(0.902-1.051) | 0.942(0.288-3.076) | - | 0.597(0.062-5.761) |
| LHR | 1.014(0.971-1.059) | - | 1.006(0.945-1.071) | 1.013(0.959-1.071) | 1.024(0.997-1.052) | **2.102(1.192-3.706)** | 3.637(0.389-33.984) | 0.666(0.194-2.291) |
| NHR | 1.011(0.974-1.049) | 0.495(0.022-11.226) | 1.008(0.959-1.061) | 1.013(0.953-1.078) | 1.010(0.978-1.043) | 1.213(0.374-3.936) | **1.934(1.027-3.643)** | 0.967(0.666-1.405) |
| Abbreviations: NLR, neutrophil to lymphocyte ratio; MLR, monocyte to lymphocyte ratio; PLR, platelet to lymphocyte ratio; CLR, hs-CRP to lymphocyte ratio; SII, neutrophil*platelet/lymphocyte; SIRI, neutrophil*monocyte/lymphocyte; AISI, neutrophil*monocyte*platelet/lymphocyte; MHR, monocyte/high-density lipoprotein cholesterol; LHR, lymphocyte/high-density lipoprotein cholesterol; NHR, neutrophil/high-density lipoprotein cholesterol.  Model adjusted for age (exclude age stratified analysis), sex (exclude sex stratified analysis), current smoking (exclude smoking stratified analysis), education level, income level, hypertension, diabetes, body mass index and family history of cancer.  -: After stratification by sex, age or smoking status the limited number of female or different age stage participants led to issues such as model non-convergence, insufficient sample size, and low event counts, among other issues. Consequently, HR (95% CI) could not be calculated for some variables. | | | | | | | | |

| Supplementary Table 9 Stratification analysis: the HR(95%CI) between inflammation and the incidence risk of Other pathological Sub-type of lung cancer | | | | | | | | |
| --- | --- | --- | --- | --- | --- | --- | --- | --- |
| Variables | Sex | | Age | | Smoking status | | | |
|  | Men | Wmen | ≥60 years old | <60 years old | Never smoking | Former smoking | Occasional smoking | Daily smoking |
| Hs-CRP | **1.063(1.008-1.122)** | 0.826(0.666-1.023) | 1.051(0.980-1.127) | 1.045(0.966-1.131) | 1.026(0.957-1.101) | 1.096(0.899-1.712) | 1.219(0.831-1.295) | 1.055(0.966-1.347) |
| WBC | **1.112(1.051-1.178)** | **1.232(1.064-1.427)** | **1.088(1.010-1.172)** | **1.166(1.078-1.260)** | **1.102(1.019-1.348)** | 1.113(0.933-4.071) | 1.092(0.792-2.841) | **1.158(1.064-1.270)** |
| Monocytes | **1.033(1.005-1.062)** | 1.035(0.938-1.142) | 1.007(0.960-1.057) | **1.051(1.018-1.084)** | **1.041(1.006-1.119)** | 0.922(0.666-3.196) | **1.227(1.091-2.952)** | 1.013(0.961-1.175) |
| Lymphocytes | **1.060(1.030-1.092)** | 1.044(0.883-1.235) | **1.064(1.014-1.117)** | **1.052(1.009-1.098)** | **1.050(1.003-1.192)** | 1.028(0.917-1.397) | **1.184(1.055-6.302)** | **1.070(1.020-1.302)** |
| Neutrophils | **1.073(1.014-1.136)** | **1.160(1.061-1.268)** | 1.055(0.977-1.140) | **1.120(1.051-1.194)** | 1.075(0.997-1.309) | 1.143(0.923-3.288) | 0.946(0.603-2.209) | **1.097(1.021-1.313)** |
| NLR | 0.946(0.855-1.046) | **1.030(1.010-1.050)** | 0.936(0.823-1.064) | 1.025(0.999-1.052) | 0.992(0.931-1.252) | 1.090(0.805-1.177) | 0.353(0.076-5.942) | 0.993(0.875-1.382) |
| MLR | 1.005(0.976-1.035) | 1.013(0.998-1.028) | 1.019(0.995-1.045) | 0.990(0.932-1.051) | **1.014(1.001-1.162)** | 0.168(0.008-3.509) | 1.093(0.949-1.258) | 0.817(0.517-1.235) |
| PLR | 0.861(0.646-1.148) | 0.997(0.979-1.016) | 0.927(0.639-1.345) | 0.912(0.669-1.242) | 0.997(0.957-1.967) | 0.691(0.313-21.213) | - | 0.811(0.572-1.330) |
| CLR | 1.017(0.991-1.044) | 1.014(0.833-1.233) | 1.020(0.988-1.053) | 1.025(0.991-1.061) | 1.012(0.967-0.981) | 1.057(0.975-0.598) | 0.689(0.314-1.444) | 1.026(0.990-1.201) |
| SII | 1.000(0.951-1.053) | 1.003(0.993-1.014) | 0.995(0.942-1.051) | 1.016(0.996-1.037) | 1.004(0.991-1.440) | 1.044(0.736-4.975) | 0.382(0.056-3.720) | 1.015(0.879-1.443) |
| SIRI | **1.616(1.115-2.341)** | **1.658(1.174-2.342)** | **2.573(1.797-3.685)** | **1.317(1.016-1.708)** | **1.636(1.262-2.191)** | - | 1.577(0.744-3.343) | 1.367(0.630-6.698) |
| AISI | 1.045(0.984-1.109) | 1.094(0.930-1.287) | 0.998(0.920-1.083) | **1.110(1.030-1.198)** | 1.061(0.973-1.380) | 0.908(0.736-2.768) | 1.297(0.959-2.449) | 1.055(0.973-1.355) |
| MHR | 1.021(0.989-1.054) | 1.010(0.996-1.024) | **1.082(1.005-1.166)** | 0.997(0.966-1.029) | **1.014(1.000-1.051)** | 0.498(0.024-3.076) | 1.105(0.979-1.248) | 1.022(0.915-5.761) |
| LHR | 1.013(0.994-1.031) | 1.006(0.979-1.035) | 1.010(0.993-1.028) | 1.012(0.987-1.037) | 1.002(0.979-1.052) | 0.958(0.693-3.706) | 1.034(0.974-33.984) | 1.025(0.999-2.291) |
| NHR | 1.006(0.989-1.024) | **1.049(1.015-1.084)** | 1.006(0.989-1.024) | 1.021(0.997-1.045) | 1.000(0.976-1.043) | 0.992(0.904-3.936) | 0.665(0.224-3.643) | **1.037(1.003-1.405)** |
| Abbreviations: NLR, neutrophil to lymphocyte ratio; MLR, monocyte to lymphocyte ratio; PLR, platelet to lymphocyte ratio; CLR, hs-CRP to lymphocyte ratio; SII, neutrophil*platelet/lymphocyte; SIRI, neutrophil*monocyte/lymphocyte; AISI, neutrophil*monocyte*platelet/lymphocyte; MHR, monocyte/high-density lipoprotein cholesterol; LHR, lymphocyte/high-density lipoprotein cholesterol; NHR, neutrophil/high-density lipoprotein cholesterol.  Model adjusted for age (exclude age stratified analysis), sex (exclude sex stratified analysis), current smoking (exclude smoking stratified analysis), education level, income level, hypertension, diabetes, body mass index and family history of cancer.  -: After stratification by sex, age or smoking status the limited number of female or different age stage participants led to issues such as model non-convergence, insufficient sample size, and low event counts, among other issues. Consequently, HR (95% CI) could not be calculated for some variables. | | | | | | | | |

| Supplementary Table 10 The HR(95%CI) between inflammation and the 10 years incidence risk of lung cancer. | | | | | |
| --- | --- | --- | --- | --- | --- |
| Variables | Lung cancer | Lung squamous cell carcinomas | Lung adenocarcinoma | Small cell lung cancer | Other pathological Sub-type of lung cancer |
| Hs-CRP | 1.014(0.956-1.076) | 1.112(0.979-1.264) | 0.903(0.799-1.021) | 0.915(0.759-1.103) | 1.042(0.989-1.099) |
| WBC | **1.128(1.064-1.195)** | 1.093(0.950-1.257) | 1.086(0.976-1.209) | 1.076(0.916-1.264) | **1.123(1.065-1.185)** |
| Monocytes | **1.072(1.045-1.101)** | **1.099(1.058-1.141)** | 1.016(0.921-1.121) | 1.047(0.999-1.097) | **1.036(1.008-1.064)** |
| Lymphocytes | 1.046(0.998-1.096) | 1.039(0.963-1.121) | 1.002(0.922-1.088) | 1.077(0.983-1.178) | **1.059(1.027-1.091)** |
| Neutrophils | **1.091(1.031-1.153)** | 1.065(0.938-1.209) | 1.077(0.980-1.183) | 1.065(0.916-1.239) | **1.084(1.030-1.141)** |
| NLR | 0.998(0.938-1.061) | 1.010(0.942-1.083) | 0.987(0.882-1.104) | 0.942(0.747-1.188) | 0.988(0.923-1.057) |
| MLR | 1.017(0.993-1.042) | **1.026(1.014-1.038)** | 1.002(0.962-1.044) | 0.948(0.796-1.130) | 1.011(0.995-1.026) |
| PLR | 1.004(0.961-1.049) | 0.908(0.564-1.461) | 1.007(0.995-1.019) | 0.995(0.837-1.183) | 0.905(0.710-1.154) |
| CLR | **1.039(1.011-1.068)** | **1.066(1.038-1.095)** | 1.027(0.953-1.107) | 0.651(0.432-0.981) | 1.019(0.993-1.045) |
| SII | 1.009(0.978-1.041) | 1.008(0.965-1.053) | 1.005(0.988-1.023) | 0.987(0.799-1.219) | 1.003(0.987-1.020) |
| SIRI | **1.754(1.350-2.278)** | **1.795(1.361-2.368)** | 0.482(0.064-3.604) | 0.973(0.428-2.211) | **1.642(1.266-2.130)** |
| AISI | **1.090(1.029-1.154)** | 1.142(1.009-1.293) | 1.056(0.950-1.174) | 1.091(0.934-1.275) | 1.050(0.993-1.111) |
| MHR | 1.018(0.998-1.039) | **1.026(1.014-1.039)** | 0.897(0.666-1.207) | 0.953(0.846-1.074) | 1.015(1.001-1.028) |
| LHR | 0.998(0.934-1.066) | 0.988(0.908-1.076) | 0.890(0.698-1.134) | 1.010(0.968-1.053) | 1.010(0.995-1.026) |
| NHR | 1.008(0.969-1.049) | 1.001(0.945-1.061) | 0.986(0.912-1.066) | 1.006(0.965-1.048) | 1.009(0.995-1.022) |
| Abbreviations: NLR, neutrophil to lymphocyte ratio; MLR, monocyte to lymphocyte ratio; PLR, platelet to lymphocyte ratio; CLR, hs-CRP to lymphocyte ratio; SII, neutrophil*platelet/lymphocyte; SIRI, neutrophil*monocyte/lymphocyte; AISI, neutrophil*monocyte*platelet/lymphocyte; MHR, monocyte/high-density lipoprotein cholesterol; LHR, lymphocyte/high-density lipoprotein cholesterol; NHR, neutrophil/high-density lipoprotein cholesterol.  Model adjusted for age, sex, current smoking, education level, income level, hypertension, diabetes, body mass index, family history of cancer . | | | | | |
